# Socioeconomic Disparities in Caregiver Burden Among Families of Older Patients With Cancer

**DOI:** 10.1001/jamahealthforum.2025.5614

**Published:** 2025-12-26

**Authors:** Wen Ju, Hao Jiang, Shanrui Ma, Bingfeng Han, Siwei Zhang, Minghua Cong, Wenqiang Wei

**Affiliations:** 1 National Central Cancer Registry, National Cancer Center/National Clinical Research Center for Cancer/Cancer Hospital, Chinese Academy of Medical Sciences and Peking Union Medical College, Beijing, China; 2 Department of Orthopedics, Chinese General PLA Hospital, Beijing, China; 3 National Clinical Research Center for Orthopedics, Sports Medicine and Rehabilitation, Beijing, China; 4 Department of Comprehensive Oncology, National Cancer Center/National Clinical Research Center for Cancer/Cancer Hospital, Chinese Academy of Medical Sciences and Peking Union Medical College, Beijing, China

## Abstract

**Importance:**

Population aging and rising cancer burden in low- and middle-income countries have intensified reliance on family caregivers, yet evidence on socioeconomic disparities in caregiving burden remains scarce.

**Objective:**

To quantify multidimensional caregiving burden, psychological distress, and economic burden, as well as identify risk factors, with a focus on the impact of socioeconomic disparities among family caregivers of older patients with cancer in China.

**Design, Setting, and Participants:**

This multicenter cross-sectional study recruited 6786 patient-caregiver dyads from 22 hospitals across 14 Chinese provinces between August 2022 and August 2023. Patients were 60 years or older with cancer; caregivers were family members or cohabiting partners 18 years or older who provided 50% or more daily care for 30 or more days. Data collection included sociodemographics, health assessments, caregiving details, expenditures, and clinical records.

**Exposures:**

Socioeconomic status (SES), categorized into 4 levels based on education and household income.

**Main Outcomes and Measures:**

Caregiving burden (measured by the Caregiver Reaction Assessment across 5 domains), psychological distress (measured by the Patient Health Questionnaire 9 and Generalized Anxiety Disorder 7 scales), and economic burden (direct caregiving expenditure and income loss).

**Results:**

Of the 6786 patient-caregiver dyads enrolled, the mean (SD) age of patients was 69.2 (6.1) years and 4128 (60.8%) were male, while the mean (SD) age of family caregivers was 53.8 (12.6) years and 3040 (44.8%) were male. Caregivers reported the following mean (SD) scores on a 5-point scale of Caregiver Reaction Assessment: health burden, 2.02 (0.69); scheduling burden, 2.64 (0.72); financial pressure, 3.03 (0.74); lack of family support, 2.09 (0.56); and self-esteem, 2.39 (0.42). Lower SES was independently associated with elevated burden across domains of health problems (β coefficient, −0.07; 95% CI, −0.10 to −0.04; *P* < .001), self-esteem (β coefficient, −0.15; −0.18 to −0.12; *P* < .001), disrupted schedule (β coefficient, −0.07; 95% CI, −0.11 to −0.03; *P* < .001), financial pressure (β coefficient, −0.14; 95% CI, −0.17 to −0.11; *P* < .001), and lack of family support (β coefficient, −0.14; 95% CI, −0.17 to −0.11; *P* < .001). Caregivers with health problems, insufficient sleep, longer care periods, lack of cocaregivers, limited nursing skills, and being employed experienced higher burden. Caregivers with the lowest level of SES faced elevated risks of anxiety (odds ratio, 1.37; 95% CI, 1.15-1.62) and depression (odds ratio, 1.71; 95% CI, 1.41-2.05) compared to caregivers with the highest level of SES. Subgroup analyses revealed that spouses reported higher health and financial burden, while higher-educated adult child caregivers were at greater risks for psychological distress. Caregivers covered 57.4% of total expenditure for older patients with cancer, and 31.7% of caregivers’ expenses for patients in the past year had exceeded their annual household income. Paradoxically, caregivers with higher SES experienced greater employment-related costs due to prolonged work absences.

**Conclusions and Relevance:**

Results of this cross-sectional study show that socioeconomic disparities profoundly shape caregiving burden, psychological distress, and economic burden faced by family caregivers of older patients with cancer. Policy interventions, targeted subsidies for families with low SES, workplace protections for employed caregivers, and integrated community support are urgently needed to address inequities and strengthen eldercare systems amid rapid population aging.

## Key Points

**Question:**

What are the socioeconomic disparities in caregiving burden, psychological distress, and economic burden faced by family caregivers of older patients with cancer?

**Findings:**

In this cross-sectional study of 6786 patient-caregiver dyads in China, lower socioeconomic status was associated with higher caregiving burden across domains of health problems, self-esteem, financial pressure, disrupted schedule, and lacking family support, as well as higher risks of depression and anxiety. Nearly one-third of caregivers spent more than their annual household income on patient expenses, with considerable disparities by socioeconomic status.

**Meaning:**

Socioeconomic disparities can exacerbate multidimensional caregiver burden; policy interventions, targeted subsidies, workplace protections, and community support are needed to mitigate caregiving strain amid aging populations.

## Teaser

This cross-sectional study aims to quantify multidimensional caregiving burden, psychological distress, and economic burden, with a focus on the impact of socioeconomic disparities, among family caregivers of older patients with cancer in China.

## Introduction

Cancer, an age-related disease, exhibits rising global incidence and mortality rates as populations age.^1^ Older patients often face comorbidities and functional impairments, necessitating substantial supportive care.^2,3^ With improved survival rates, the care needs of older patients have become a growing concern for policymakers. Informal care, primarily provided by family members, is crucial in current health care systems. These caregivers assist with treatment decisions, daily activities, and emotional and financial support, yet they are more susceptible to health issues, psychological distress, and financial strain compared to noncaregivers.^4,5,6^ However, caregivers’ experiences in rapidly aging low- and middle-income countries remain underexplored.

Socioeconomic status (SES) considerably influences health care access and outcomes.^7,8,9^ In high-income countries, disparities in care provision emerge as costly formal care systems often fail to address comprehensive needs, leading to fragmented integration of formal and informal care services.^10^ Patients with lower SES and limited education and income rely more on family care due to inadequate access to medical and institutional support.^11,12^ However, research on socioeconomic disparities in caregiver burden is still scarce and urgently needed for health care policy development.

China, with the world’s largest older population, reports millions of age-related cancer cases annually.^13^ Unlike high-income nations with robust health care infrastructure,^14,15^ eldercare in China remains a familial obligation rooted in cultural tradition.^16^ Existing studies on caregiver burden in China are predominantly limited to single-center, small-sample designs focusing on isolated burden dimensions.^17,18^ To sustain caregivers’ critical roles, policymakers must prioritize understanding and mitigating their multifaceted challenges. This necessitates systematic research to comprehensively assess caregiving-related details and burden, identifying potential risks.

The Chinese Caregiver of Older Cancer Patient study is a multicenter observational investigation designed to systematically document the comprehensive caregiving-related details and quantify the multidimensional caregiving burden, psychological distress, and economic burden among family caregivers, with a focus on the impact of socioeconomic disparities through standardized metrics. Analyses were stratified by caregiver type (spouse vs adult child), aiming to enhance understanding of caregiver challenges and inform equitable policies and interventions to mitigate caregiving strain amid aging populations.

## Methods

### Study Design and Participants

The baseline survey of the Chinese Caregiver of Older Cancer Patient study was conducted in 22 large public hospitals and specialized cancer centers across 14 provinces in China, covering eastern, central, and western regions (eTable 1 and eFigure 1 in Supplement 1). Within each hospital, 2 to 4 wards from oncology departments (surgery, medical oncology, and radiotherapy) were randomly selected as sampling units.

Patient-caregiver dyads were included based on the following criteria. Patients were eligible if they (1) were 60 years or older with a clinically confirmed cancer, (2) were hospitalized in participating centers between August 2022 and August 2023, (3) had adequate cognitive capacity to provide informed consent and complete assessments, and (4) provided written informed consent. Caregivers were eligible if they (1) were the primary informal caregiver (family member or cohabiting partner aged ≥18 years) providing 50% or more of daily care for 30 or more consecutive days, (2) had intact consciousness and communication abilities, and (3) provided voluntary informed consent. The process of participant selection is depicted in eFigure 2 in Supplement 1.

Written informed consent was obtained from all participants or their legal representatives. The study was approved by the ethics committee of the National Cancer Center/National Clinical Research Center for Cancer/Cancer Hospital of the Chinese Academy of Medical Sciences and Peking Union Medical College. We followed the Strengthening the Reporting of Observational Studies in Epidemiology (STROBE) reporting guidelines.

### Data Collection

Trained medical staff conducted face-to-face surveys using an electronic questionnaire to collect data on sociodemographics, health and nutrition status, caregiving-related details, expenditure data, and scales for caregiver burden and depression/anxiety symptoms. Clinical data were extracted from medical records. Patient health and nutrition measurements were taken by physicians or nurses during hospitalization. Additional information is provided in the eMethods in Supplement 1. Quality assessments ensured data completeness.

### Exposure

The SES index integrated caregivers’ household annual income and education. Education was categorized into primary (middle school or below), secondary (high school), or tertiary (college or above); household income was categorized into quartiles: less than ¥20 000 (US $2814), ¥20 000 to ¥59 999 (US $2814-$8441), ¥60 000 to ¥99 999 (US $8441-$14 069), and ¥100 000 (US $14 069) or higher. Caregivers were categorized into 4 SES groups: lowest, lower middle, upper middle, and highest. The detailed SES construction is introduced in the eMethods in Supplement 1.

### Outcomes

Caregiving-related burden was assessed using the Caregiver Reaction Assessment (CRA) scale, encompassing 5 subscales: health problems, disrupted schedule, financial pressure, lack of family support, and self-esteem.^19^ Depression and anxiety symptoms were assessed using the Patient Health Questionnaire 9 and the Generalized Anxiety Disorder 7 scales, with a score of 5 or higher indicating depressive or anxiety symptoms.

Economic burden included direct caregiving costs and income loss. Direct costs included annual medical and nonmedical expenses (eg, transportation, lodging, nutrition, nursing). The expense to income ratio was calculated as annual caregiving expenses divided by annual household income. Caregivers’ income loss refers to the reduced earnings due to work absences, tardiness, or early departures caused by caregiving. Employed caregivers reported days absent and income lost due to caregiving responsibilities in the past month, including lost full-attendance bonuses. Details are in the eMethods in Supplement 1.

### Statistical Analysis

Characteristics were stratified by SES and presented as percentages (categorical variables) or mean (SD) and median (IQR) (continuous variables). Trends across SES groups were tested using analysis of variance or Mantel-Haenszel χ^2^ tests. CRA subscale scores were summarized by SES, education, and income levels. Generalized linear models estimated associations between SES, education, income, and CRA scores, and were adjusted for caregiver demographics (sex and age), caregiver factors (self-reported health problems, employment, sleep, care days, cocaregivers, and nursing skill), and patient factors (age, cancer stage, surgery, prevalent years, Nutritional Risk Screening 2002 score, and EQ-5D-5L score for quality of life). Multivariable logistic regression models assessed risk of anxiety and depression by SES, education, and household income, and were adjusted for the previously described covariates.

In sensitivity analyses, we further adjusted for the Social Deprivation Index (SDI; eTable 2 and eMethods in Supplement 1) to assess the robustness of the multivariate models. The potential interaction between education and household income was assessed using stratified analyses. The statistical significance of the interaction was evaluated with the likelihood ratio test, which compares regression models with and without the interaction term. All analyses were stratified by caregiver type (spouse vs adult child) a priori, as cultural expectations, caregiving roles, and socioeconomic dynamics may differentially influence the burden and experiences of these 2 distinct caregiver groups.^20^ Group differences in caregiving expenses were analyzed using the Kruskal-Wallis H test, Mann-Whitney U test, and rank correlation tests. Spearman correlation tests were used to analyze associations between SES score and work days/income loss. Analyses were conducted using SAS, version 9.4 (SAS Institute Inc), and 2-sided *P* < .05 was considered statistically significant.

## Results

### Study Population

A total of 6786 patient-caregiver dyads were enrolled between August 2022 and August 2023. Of the patients (mean [SD] age, 69.2 [6.1] years; 4128 [60.8%] male), 2320 (34.1%) were diagnosed with lung cancer, 967 (14.2%) with colorectal cancer, 558 (8.2%) with gastric cancer, 545 (8.0%) with esophageal cancer, and 453 (6.7%) with breast cancer. Additionally, 5077 patients (74.8%) had advanced-stage (III/IV) disease, with 3816 (56.2%) diagnosed within the past year and 1216 (17.9%) more than 3 years prior (Table 1).

**Table 1.** Characteristics of Older Patients With Cancer by Socioeconomic Status Quartiles

Characteristic	No. (%)					*P* value
	Overall (N = 6786)	Lowest (n = 1872)	Lower middle (n = 1739)	Upper middle (n = 1320)	Highest (n = 1712)	
Age, mean (SD), y	69.2 (6.1)	68.6 (5.6)	69.3 (6.0)	69.3 (6.0)	69.5 (6.6)	<.001
Sex						
Female	2658 (39.2)	706 (37.7)	681 (39.2)	497 (37.7)	716 (41.8)	.03
Male	4128 (60.8)	1166 (62.3)	1058 (60.8)	823 (62.3)	996 (58.2)	
Children						
0	55 (0.8)	21 (1.1)	11 (0.6)	12 (0.9)	10 (0.6)	<.001
1	2017 (29.7)	491 (26.2)	537 (30.9)	411 (31.1)	531 (31)	
2	2834 (41.8)	779 (41.6)	685 (39.4)	554 (42)	758 (44.3)	
≥3	1839 (27.1)	579 (30.9)	491 (28.2)	331 (25.1)	401 (23.4)	
Missing	41 (0.6)	2 (0.1)	15 (0.9)	12 (0.9)	12 (0.7)	
Comorbidity						
No	3743 (55.2)	1011 (54.0)	967 (55.6)	688 (52.1)	991 (57.9)	.10
Yes	3043 (44.8)	861 (46.0)	772 (44.4)	632 (47.9)	721 (42.1)	
Cancer site						
Lung	2320 (34.2)	642 (34.3)	603 (34.7)	437 (33.1)	624 (36.4)	.008
Stomach	558 (8.2)	187 (10.0)	139 (8.0)	106 (8.0)	118 (6.9)	
Esophagus	545 (8.0)	177 (9.5)	125 (7.2)	113 (8.6)	123 (7.2)	
Colorectum	967 (14.2)	277 (14.8)	235 (13.5)	215 (16.3)	227 (13.3)	
Liver	308 (4.5)	75 (4.0)	70 (4.0)	65 (4.9)	92 (5.4)	
Breast	453 (6.7)	101 (5.4)	156 (9.0)	68 (5.2)	122 (7.1)	
Other	1492 (21.9)	399 (21.3)	382 (22.0)	296 (22.5)	393 (23.0)	
Missing	143 (2.1)	14 (0.7)	29 (1.7)	20 (1.5)	13 (0.8)	
Time prevalent, y						
<1	3816 (56.2)	972 (51.9)	952 (54.7)	725 (54.9)	1053 (61.5)	<.001
1-2.9	1754 (25.8)	534 (28.5)	450 (25.9)	366 (27.7)	389 (22.7)	
3-4.9	601 (8.9)	195 (10.4)	159 (9.1)	112 (8.5)	128 (7.5)	
≥5	615 (9.1)	171 (9.1)	178 (10.2)	117 (8.9)	142 (8.3)	
First diagnosis						
Yes	317 (4.7)	48 (2.6)	73 (4.2)	1264 (95.8)	1645 (96.1)	.04
No	6469 (95.3)	1824 (97.4)	1666 (95.8)	56 (4.2)	67 (3.9)	
Stage						
0	80 (1.2)	39 (2.1)	22 (1.3)	11 (0.8)	5 (0.3)	<.001
I	288 (4.2)	83 (4.4)	59 (3.4)	62 (4.7)	82 (4.8)	
II	565 (8.3)	169 (9.0)	155 (8.9)	99 (7.5)	132 (7.7)	
III	1483 (21.9)	446 (23.8)	353 (20.3)	309 (23.4)	358 (20.9)	
IV	3594 (53.0)	927 (49.5)	993 (57.1)	683 (51.7)	956 (55.8)	
Missing	776 (11.4)	208 (11.1)	157 (9.0)	156 (11.8)	179 (10.5)	
Hospital stay, d						
≤3	1860 (27.4)	447 (23.9)	520 (29.9)	336 (25.5)	480 (28.0)	.03
4 to <7	2571 (37.9)	762 (40.7)	650 (37.4)	490 (37.1)	646 (37.7)	
7 to 10	967 (14.2)	264 (14.1)	218 (12.5)	201 (15.2)	265 (15.5)	
>10	1388 (20.5)	399 (21.3)	351 (20.2)	293 (22.2)	321 (18.8)	
Surgical treatment						
Yes	492 (7.3)	82 (4.4)	127 (7.3)	123 (9.3)	148 (8.6)	<.001
No	6151 (90.6)	1776 (94.9)	1583 (91.0)	1177 (89.2)	1551 (90.6)	
Missing	143 (2.1)	14 (0.7)	29 (1.7)	20 (1.5)	13 (0.8)	
NRS 2002 score						
<3	4159 (61.3)	1166 (62.3)	1131 (65.0)	780 (59.1)	1013 (59.2)	.005
≥3	2627 (38.7)	706 (37.7)	608 (35.0)	540 (40.9)	699 (40.8)	
SARC-F score						
<4	4685 (69.0)	1386 (74.0)	1147 (66.0)	904 (68.5)	1135 (66.3)	<.001
≥4	2101 (31.0)	486 (26.0)	592 (34.0)	416 (31.5)	577 (33.7)	
EQ-5D-5L score, mean (SD)	0.83 (0.23)	0.87 (0.20)	0.83 (0.22)	0.83 (0.24)	0.80 (0.24)	<.001
Grip per kg, mean (SD)	21.6 (7.5)	21.3 (8.2)	22.0 (7.1)	22.1 (7.3)	20.9 (7.1)	.59
Calf circumference, mean (SD), cm	33.0 (4.7)	33.4 (8.2)	32.8 (4.5)	32.7 (4.5)	33.1 (4.4)	.64
Arm circumference, mean (SD), cm	27.0 (4.5)	27.8 (4.8)	26.5 (4.3)	26.8 (4.3)	26.5 (4.4)	<.001
BMI, mean (SD)	22.3 (3.5)	22.2 (3.5)	22.3 (3.6)	22.4 (3.5)	22.2 (3.3)	.52

Abbreviations: BMI, body mass index (calculated as weight in kilograms divided by height in meters squared); NRS 2002, Nutritional Risk Screening 2002; SARC-F, Strength, Assistance With Walking, Rise From a Chair, Climb Stairs, and Falls.

Of the of 6786 family caregivers (mean [SD] age, 53.8 [12.6] years; 3040 [44.8%] male), 3404 (50.2%) were adult children and 2812 (41.4%) were spouses. Education levels of caregivers included 3906 (57.6%) with middle school and below, 2165 (31.9%) with high school, and 701 (10.3%) with college or above. Across annual household incomes, 737 caregivers (10.9%) earned less than ¥20 000, 1662 (24.5%) earned ¥20 000 to ¥59 999, 2180 (32.1%) earned ¥60 000 to ¥99 999, and 2078 (31.2%) earned ¥100 000 or more. Caregivers with lower SES were more often unemployed; had fewer cocaregivers, sleep time, and nursing-care skills; and had more health problems (Table 2).

**Table 2.** Characteristics of Family Caregivers by Socioeconomic Status Quartiles

Characteristic	No. (%)						*P* value
	Overall (N = 6786)	Lowest (n = 1872)	Lower middle (n = 1739)	Upper middle (n = 1320)	Highest (n = 1712)		
Sex							
Female	3618 (53.3)	1132 (60.5)	974 (56.0)	685 (51.9)		821 (48.0)	<.001
Male	3040 (44.8)	740 (39.5)	765 (44.0)	635 (48.1)		891 (52.0)	
Missing	128 (1.9)	0	0	0		0	
Age, mean (SD), y	53.8 (12.6)	57.9 (11.8)	55.1 (12.3)	52.4 (12.4)		48.9 (12.1)	<.001
Age group, y							
<60	4038 (59.5)	822 (43.9)	960 (55.2)	826 (62.6)		1291 (75.4)	<.001
≥60	2748 (40.5)	1050 (56.1)	779 (44.8)	494 (37.4)		421 (24.6)	
Marital status							
Single	179 (2.6)	52 (2.8)	37 (2.1)	37 (2.8)		53 (3.1)	.35
Married	6479 (95.5)	1820 (97.2)	1702 (97.9)	1283 (97.2)		1659 (96.9)	
Missing	128 (1.9)	0	0	0		0	
Family income per year, ¥							
<20 000 (US $2814)	737 (10.9)	NA	NA	NA		NA	NA
20 000-59 999 (US $2814-$8441)	1662 (24.5)	NA	NA	NA		NA	
60 000-99 999 (US $8441-$14 069)	2180 (32.1)	NA	NA	NA		NA	
≥100 000 (US $14 069)	2078 (31.2)	NA	NA	NA		NA	
Missing	129 (1.9)	NA	NA	NA		NA	
Education							
Middle school and below	3906 (57.6)	NA	NA	NA		NA	NA
High school	2165 (31.9)	NA	NA	NA		NA	
College and above	701 (10.3)	NA	NA	NA		NA	
Missing	14 (0.2)	NA	NA	NA		NA	
Work status							
Employed	3219 (47.4)	566 (30.2)	747 (43.0)	705 (53.4)		1192 (69.6)	<.001
Unemployed	3439 (50.7)	1306 (69.8)	992 (57.0)	615 (46.6)		520 (30.4)	
Missing	128 (1.9)	0	0	0		0	
Relationship with patient							
Spouse	2812 (41.4)	1125 (60.1)	792 (45.5)	494 (37.4)		396 (23.1)	<.001
Child	3404 (50.2)	645 (34.5)	835 (48.0)	722 (54.7)		1193 (69.7)	
Other	441 (6.5)	102 (5.4)	112 (6.4)	104 (7.9)		123 (7.2)	
Missing	129 (1.9)	0	0	0		0	
Self-reported health problem							
No	5680 (83.7)	1453 (77.6)	1494 (85.9)	1162 (88.0)		1559 (91.1)	<.001
Yes	977 (14.4)	419 (22.4)	245 (14.1)	158 (12.0)		153 (8.9)	
Missing	129 (1.9)	0	0	0		0	
Cocaregivers							
No	3924 (57.8)	1343 (71.7)	1036 (59.6)	763 (57.8)		775 (45.3)	<.001
Yes	2733 (40.3)	529 (28.3)	703 (40.4)	557 (42.2)		937 (54.7)	
Missing	129 (1.9)	0	0	0		0	
Duration of current care							
Episode, d							
<3	1114 (16.4)	243 (13.0)	322 (18.5)	212 (16.1)		336 (19.6)	<.001
3-5	2415 (35.6)	712 (38.0)	635 (36.5)	453 (34.3)		607 (35.5)	
6-10	1951 (28.8)	560 (29.9)	473 (27.2)	422 (32.0)		496 (29.0)	
>10	1139 (16.8)	353 (18.9)	300 (17.3)	225 (17.0)		256 (15.0)	
Missing	167 (2.5)	4 (0.2)	9 (0.5)	8 (0.6)		17 (1.0)	
Caregivers’ sleep per day, h							
<6	1453 (21.4)	546 (29.2)	378 (21.7)	253 (19.2)		271 (15.8)	<.001
6-8	4652 (68.6)	1194 (63.8)	1240 (71.3)	952 (72.1)		1258 (73.5)	
>8	552 (8.1)	132 (7.1)	121 (7)	115 (8.7)		183 (10.7)	
Missing	129 (1.9)	0	0	0		0	
Nursing care skills							
Extremely skilled	135 (2.0)	12 (0.6)	20 (1.2)	36 (2.7)		67 (3.9)	<.001
Moderately skilled	2822 (41.6)	550 (29.4)	670 (38.5)	590 (44.7)		1012 (59.1)	
Not skilled at all	3686 (54.3)	1310 (70.0)	1049 (60.3)	694 (52.6)		633 (37.0)	
Missing	143 (2.1)	0	0	0		0	

Abbreviation: NA, not applicable.

### CRA and SES

Caregivers reported the following mean (SD) scores on a 5-point scale of CRA: health burden, 2.02 (0.69); scheduling burden, 2.64 (0.72); financial pressure, 3.03 (0.74); lack of family support, 2.09 (0.56); and self-esteem, 2.39 (0.42). Spouse caregivers reported statistically significant higher scores in health problems, financial pressure, self-esteem, and lack of family support subscales than adult child caregivers, though a lower score in schedule disruptions (eTable 3 in Supplement 1). Mean scores for all CRA subscales exhibited pronounced SES gradients (Figure).

**Figure.**
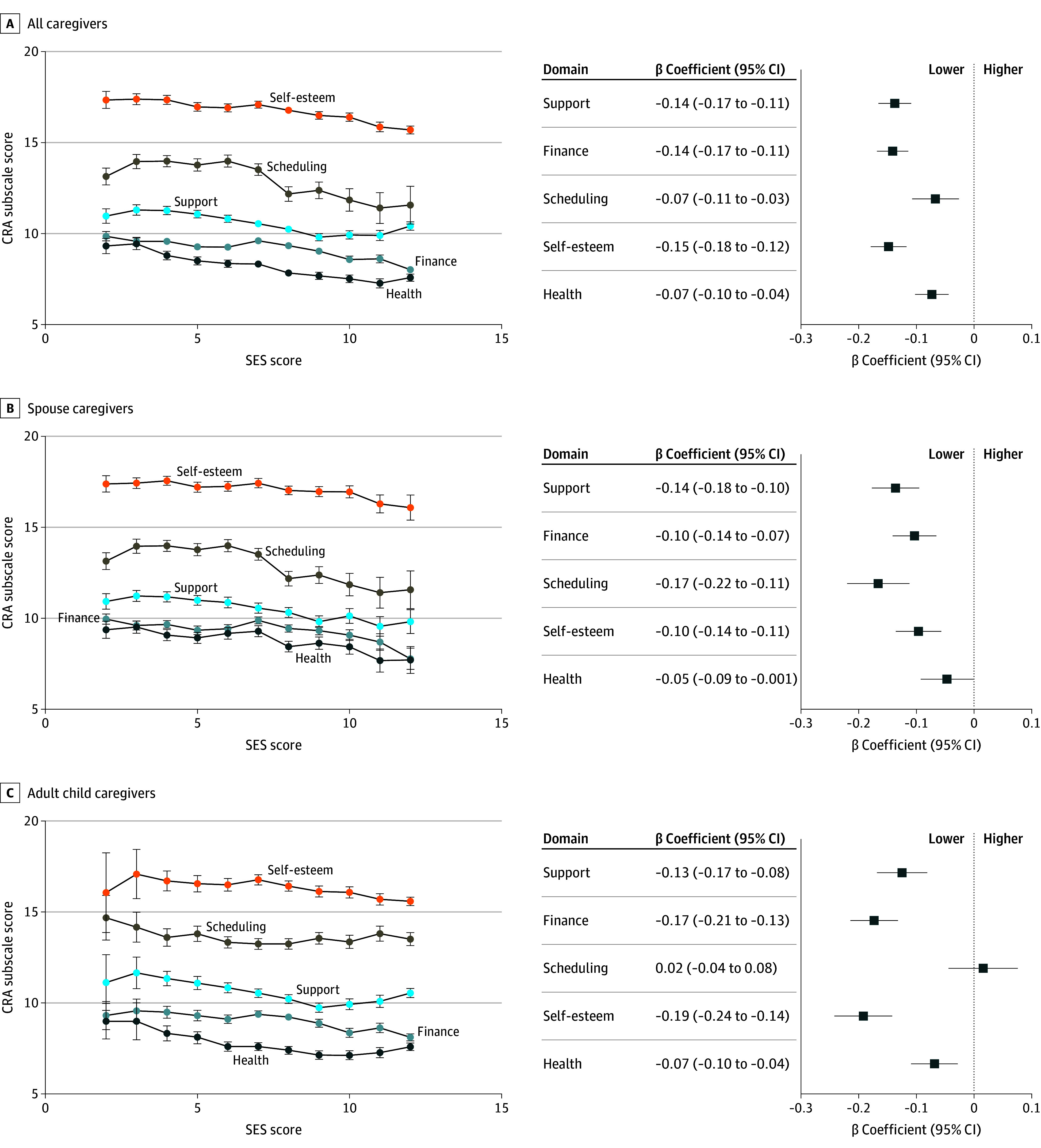
Mean Caregiver Reaction Assessment (CRA) Scores by Domain and Associations With Socioeconomic Status (SES) Error bars indicate 95% CIs.

Multivariable models confirmed SES as an independent risk factor of caregiver burden (Figure). Each 1-unit SES score increase corresponded to statistically significant reductions in caregiving burden for all caregivers across health problems (β coefficient, −0.07; 95% CI, −0.10 to −0.04; *P* < .001), self-esteem (β coefficient, −0.15; 95% CI, −0.18 to −0.12; *P* < .001), schedule disruptions (β coefficient, −0.07; 95% CI, −0.11 to −0.03; *P* < .001), financial pressure (β coefficient, −0.14; 95% CI, −0.17 to −0.11; *P* < .001), and lack of family support (β coefficient, −0.14; 95% CI, −0.17 to −0.11; *P* < .001). Stratified analyses highlighted role-specific vulnerabilities: for spouse caregivers, SES had the highest association with schedule disruptions (β coefficient, −0.17; 95% CI, −0.22 to −0.11), while adult child caregivers had highest associations of SES and self-esteem (β coefficient, −0.19; 95% CI, −0.24 to −0.14).

Additionally, female spouse caregivers reported a higher health burden, while male adult-child caregivers experienced higher scheduling and family support burdens and lower self-esteem (eTable 4 in Supplement 1). Caregivers who lacked cocaregivers, were employed, and had health problems, inadequate sleep, extended caregiving duration, and limited nursing skills had higher burdens. Conversely, caregivers for patients with early-stage cancer, surgical intervention, better nutrition, and higher EQ-5D-5L scores experienced lower caregiving-related burden (eTable 4 in Supplement 1).

A sensitivity analysis was conducted by further adjusting for the SDI in the generalized linear models. The associations between SES and caregiver burden remained largely unchanged after including the SDI covariate, indicating robust findings. Notably, higher regional SDI was independently associated with greater burden across all 5 CRA dimensions (eTable 5 in Supplement 1).

The associations of education and household income with caregiver burden, along with their interactions, are detailed in eTable 6 in Supplement 1. Lower education was associated with greater burden in finance (β coefficient, 1.09; 95% CI, 0.89-1.28) and self-esteem (β coefficient, 0.89; 95% CI, 0.63-1.14). Lower income was consistently associated with higher burden across health, financial, and family support domains. Statistically significant interactions between education and income were observed for financial burden (*P* for interaction = .02) and lack of family support (*P* for interaction < .001), underscoring their association with caregiver burden (eTable 6 in Supplement 1).

### Depression/Anxiety Symptoms and SES

The prevalence of anxiety symptoms (Generalized Anxiety Disorder 7 score ≥5) was 37.2% among all caregivers (976 [34.7%] spouses and 1393 [40.9%] adult children). Depression symptoms (Patient Health Questionnaire 9 score ≥5) affected 26.7% overall (687 [24.4%] spouses and 1018 [29.9%] adult children) (eTable 7 in Supplement 1).

There was a statistically significant association between SES and caregivers’ psychological outcomes, with pronounced gradients in anxiety and depression risks (Table 3). Caregivers in the lowest SES group faced elevated risks of anxiety (odds ratio [OR], 1.37; 95% CI, 1.15-1.62) and depression (OR, 1.71; 95% CI, 1.41-2.05) compared to the highest SES group. Subgroup analyses revealed critical disparities by caregiver role: spouse caregivers in the lowest SES exhibited markedly higher anxiety (OR, 1.59; 95% CI, 1.18-2.14) and depression (OR, 2.57; 95% CI, 1.80-3.68) risks, while adult child caregivers in the same SES stratum showed increased depression risk (OR, 1.37; 95% CI, 1.06-1.78) but reduced risk in the upper-middle SES group (OR, 0.68; 95% CI, 0.53-0.87). After further adjustment for SDI, the lowest-SES group retained increased risks of anxiety (OR, 1.22; 95% CI, 1.04-1.47) and depression (OR, 1.49; 95% CI, 1.24-1.80) compared to the highest-SES group, confirming the robustness of the primary findings (eTable 8 in Supplement 1).

**Table 3.** Associations of Socioeconomic Status (SES) Status, Educational Level, Household Income Level, and Expense to Income Ratio With Risk of Depression and Anxiety Symptoms Among Caregivers^a^

Variable	Depression symptoms (PHQ-9 score ≥5)						Anxiety symptoms (GAD-7 score ≥5)					
	All caregivers, OR (95% CI)	*P* value	Spouse caregivers, OR (95% CI)	*P* value	Adult child caregivers, OR (95% CI)	*P* value	All caregivers, OR (95% CI)	*P* value	Spouse caregivers, OR (95% CI)	*P* value	Adult child caregivers, OR (95% CI)	*P* value
SES group^b^												
Lowest	1.71 (1.42-2.05)	<.001	2.57 (1.80-3.68)	<.001	1.37 (1.06-1.78)	.02	1.37 (1.15-1.62)	<.001	1.59 (1.18-2.14)	.002	1.25 (0.98-1.61)	.08
Lower middle	0.94 (0.79-1.13)	.05	1.31 (0.90-1.90)	.17	0.93 (0.74-1.17)	.52	0.99 (0.84-1.17)	.90	1.18 (0.87-1.61)	.29	1.06 (0.85-1.31)	.62
Upper middle	0.75 (0.62-0.92)	<.001	1.14 (0.76-1.72)	.53	0.68 (0.53-0.87)	.002	0.82 (0.69-0.98)	.03	1.01 (0.72-1.42)	.93	0.82 (0.65-1.02)	.08
Highest	1 [Reference]	NA	1 [Reference]	NA	1 [Reference]	NA	1 [Reference]	NA	1 [Reference]	NA	1 [Reference]	NA
Educational level												
Primary	0.65 (0.53-0.80)	<.001	0.71 (0.38-1.32)	.28	0.63 (0.49-0.8)	<.001	0.70 (0.57-0.85)	<.001	0.72 (0.40-1.29)	.27	0.69 (0.55-0.87)	.002
Secondary	0.72 (0.59-0.88)	.002	0.76 (0.40-1.44)	.40	0.77 (0.61-0.96)	.02	0.68 (0.56-0.83)	<.001	0.69 (0.38-1.26)	.23	0.73 (0.58-0.91)	.006
Tertiary	1 [Reference]	NA	1 [Reference]	NA	1 [Reference]	NA	1 [Reference]	NA	1 [Reference]	NA	1 [Reference]	NA
Household income level, quartile^c^												
1	3.15 (2.51-3.95)	<.001	4.05 (2.88-5.71)	<.001	3.28 (2.16-4.97)	<.001	2.17 (1.75-2.68)	<.001	2.19 (1.64-2.94)	<.001	2.53 (1.69-3.79)	<.001
2	1.58 (1.32-1.88)	<.001	2.37 (1.71-3.29)	<.001	1.35 (1.06-1.71)	.14	1.20 (1.02-1.40)	.03	1.41 (1.08-1.85)	.01	1.19 (0.95-1.49)	.12
3	0.95 (0.80-1.12)	.52	1.28 (0.93-1.78)	.14	0.92 (0.74-1.13)	.42	0.97 (0.84-1.13)	.73	1.17 (0.90-1.53)	.23	1.02 (0.84-1.24)	.86
4	1 [Reference]	NA	1 [Reference]	NA	1 [Reference]	NA	1 [Reference]	NA	1 [Reference]	NA	1 [Reference]	NA
Expense to income ratio, quintile^d^												
1	7.77 (6.00-10.07)	<.001	7.98 (5.48-11.61)	<.001	6.21 (4.04-9.54)	<.001	4.59 (3.66-5.76)	<.001	4.79 (3.50-6.56)	<.001	3.28 (2.22-4.84)	<.001
2	4.84 (3.80-6.16)	<.001	4.46 (3.04-6.56)	<.001	5.43 (3.83-7.71)	<.001	3.99 (3.24-4.92)	<.001	4.24 (3.08-5.83)	<.001	3.56 (2.62-4.85)	<.001
3	3.09 (2.46-3.89)	<.001	2.86 (1.94-4.22)	<.001	3.06 (2.22-4.24)	<.001	2.85 (2.34-3.46)	<.001	3.24 (2.37-4.44)	<.001	2.21 (1.67-2.92)	<.001
4	1.38 (1.07-1.77)	.01	1.29 (0.83-2.01)	.25	1.25 (0.89-1.77)	.20	1.37 (1.11-1.68)	.003	1.29 (0.91-1.83)	.16	1.17 (0.87-1.56)	.29
5	1 [Reference]	NA	1 [Reference]	NA	1 [Reference]	NA	1 [Reference]	NA	1 [Reference]	NA	1 [Reference]	NA

Abbreviations: GAD-7, Generalized Anxiety Disorder 7; NA, not applicable; OR, odds ratio; PHQ-9, Patient Health Questionnaire 9

^a^ *P* values were estimated by multivariable logistic regression. The models were adjusted for caregiver factors (morbidity and nursing care skill) and patient factors (age group, cancer stage, surgery, prevalent years, Nutritional Risk Screening 2002 score, and EQ-5D-5L score for quality of life). The reference groups are the highest levels in SES, education, household income, and expense to income ratio.

^b^ SES is categorized into 4 groups according to the combination of household income level (1, 2, 3, or 4) and education level (1, 2, or 3): lowest SES (2-3), lower-middle SES (4), upper-middle SES (5), and highest SES (6-8).

^c^ Quartile 1 indicates less than ¥20 000 (US $2814); quartile 2, ¥20 000 to ¥59 999 (US $2814-$8441); quartile 3, ¥60 000 to ¥99 999 (US $8441-$14 069); quartile 4, ¥100 000 (US $14 069) or higher.

^d^ The annual expense to income ratio is defined as the proportion of caregiving-related expenses incurred by the caregiver relative to the caregiver’s household income in the past year. Quintile 1 indicates more than 2; quintile 2, 1 to 2; quintile 3, 0.5 to less than 1; quintile 4, 0.2 to less than 0.5; quintile 5, 0 to less than 0.2.

Lower household income was associated with independently heightened susceptibility to anxiety (OR, 2.17; 95% CI, 1.75-2.68) and depression (OR, 3.15; 95% CI, 2.51-3.95) among all caregivers. Conversely, lower education (primary vs tertiary) among adult child caregivers was associated with reduced anxiety (OR, 0.69; 95% CI, 0.55-0.87) and depression (OR, 0.63; 95% CI, 0.49-0.80) risks (Table 3). A statistically significant association between education and income was observed for both depression (*P* for interaction = .007) and anxiety (*P* for interaction < .001) (eTable 9 in Supplement 1). These stratified analyses revealed that lower education was associated with a lower risk of depression and anxiety, which was most pronounced in the highest-income quartile, where primary education was associated with substantially reduced risks of depression (OR, 0.47; 95% CI, 0.34-0.64) and anxiety (OR, 0.27; 95% CI, 0.19-0.40) compared to tertiary education (eTable 9 in Supplement 1). Additionally, the expense to income ratio demonstrated a dose-response association, with caregivers in the highest-burden quintile (ratio, >2) experiencing higher risks of anxiety (OR, 7.77; 95% CI, 6.00-10.07) and depression (OR, 4.59; 95% CI, 3.66-5.76) compared to the lowest burden group (ratio, <0.2) (*P* < .001) (Table 2).

### Direct Caregiving-Related Cost for Caregivers

Patients with cancer incurred substantial annual medical expenditures, averaging ¥100 694 (US $14 176) (median, ¥88 080 [US $12 400]) (eTable 10 in Supplement 1). Family caregivers bore 57.4% of this financial burden at a median (IQR) cost of ¥44 980 (¥17 600-¥82 420) (US $6328 [US $2476-$11 595]), with spouse caregivers contributing a higher proportion than adult child caregivers (60.8% vs 57.7%; *P* < .001) (Table 4 and eFigure 3 in Supplement 1). Direct medical treatment constituted the largest expense category at 78.7% and a median (IQR) cost of ¥30 000 (¥6000-¥65 125) (US $4221 [US $875-9513]), followed by hospitalization and transportation costs. Costs for nutritional support and professional nursing were minimal, indicating limited access to ancillary care services.

**Table 4.** Caregiving-Related Expenses by Family Caregivers in the Past Year^a^^,^^b^

Variable	Total expense		Medical expense		Expense for transportation		Expense for board and lodging at hospitals		Expense for nutrition^c^		Expense for nursing staff^c^		Expense to income ratio^d^	
	Median (IQR), ¥	*P* value	Median (IQR), ¥	*P* value	Median (IQR), ¥	*P* value	Median (IQR), ¥	*P* value	Median (IQR), ¥	*P* value	Median (IQR), ¥	*P* value	Expense to income ratio >1, No. (%)	*P* value
Overall	44 980 (17 600-82 420)	NA	30 000 (6000-65 125)	NA	1200 (480-4200)	NA	4800 (2400-9000)	NA	0 (0-1800)	NA	0 (0-0)	NA	1929 (31.7)	NA
Relationship														
Spouse	43 120 (13 920-84 800)	<.001	30 000 (900-70 000)	<.001	960 (240-2400)	<.001	4800 (2400-8400)	<.001	0 (0-0)	<.001	0 (0-0)	.007	999 (37.8)	<.001
Adult child	49 200 (25 097-86 120)		35 275 (13 350-70 000)		2400 (720-4800)		6000 (3000-9600)		0 (0-2910)		0 (0-0)		888 (28.7)	
Other	9740 (0-38 800)		0 (0-25 000)		480 (0-2400)		2400 (0-6720)		0 (0-0)		0 (0-0)		48 (12.8)	
Cocaregivers														
No	50 800 (21 320-86 240)	<.001	38 000 (8000-70 000)	<.001	1440 (480-4800)	<.001	6000 (3600-9600)	<.001	0 (0-0)	<.001	0 (0-0)	.35	1400 (38.9)	<.001
Yes	36 200 (14 328-77 680)		25 641 (4500-60 000)		1200 (360-3600)		4080 (1800-7200)		0 (0-2400)		0 (0-0)		529 (21.2)	
SES group														
Lowest	41 600 (15 840-75 960)	<.001	30 000 (3100-60 000)	<.001	1920 (480-4800)	<.001	6000 (2880-9600)	<.001	0 (0-258)	<.001	0 (0-0)	<.001	993 (57.2)	<.001
Lower middle	39 600 (13 500-72 000)		30 000 (3600-60 000)		1200 (360-3600)		4800 (1800-7200)		0 (0-480)		0 (0-0)		502 (30.3)	
Upper middle	42 960 (15 900-76 000)		30 000 (5000-60 000)		1200 (360-3600)		4800 (2400-8400)		0 (0-720)		0 (0-0)		223 (18.0)	
Highest	59 600 (26 400-106 080)		48 000 (15 500-88 763)		1440 (480-4800)		4800 (2400-9600)		0 (0-4200)		0 (0-0)		207 (14.3)	
HDI of regions^e^														
Low	45 600 (20 400-79 760)	.09	32 000 (9860-63 406)	.20	1200 (600-4788)	.003	6000 (3600-9600)	<.001	0 (0-2400)	<.001	0 (0-0)	.51	482 (26.5)	<.001
Middle	44 900 (16 800-80 100)		30 300 (4420-63 500)		1440 (300-3840)		5160 (2400-8400)		0 (0-0)		0 (0-0)		988 (36.1)	
High	44 052 (15 900-90 700)		30 000 (7100-72 000)		1200 (480-3840)		3840 (1560-7200)		0 (0-3600)		0 (0-0)		458 (29.8)	

Abbreviations: HDI, High Development Index; NA, not appliable; SES, socioeconomic status.

^a^ The exchange rate for Chinese yuan renminbi to US dollars is 1 to 0.14 as of November 20, 2025.

^b^ The difference among groups was explored by Kruskal-Wallis H test. The differences among groups by age and cocaregiver status were explored by Mann-Whitney U test. The differences between levels of education, household income, socioeconomic status, HDI of regions, and expense were explored by rank correlation tests.

^c^ For expense categories where a substantial proportion of caregivers reported no cost (median, 0), the proportion of caregivers with any expense (>¥0) and the median (IQR) among those caregivers are as follows: expense for nutrition, 28.2% (n = 1756) and ¥5400 (¥3000-¥9600) (US $764 [US $424-$1358]); expense for nursing staff, 0.5% (n = 32) and ¥12 900 (¥11 940-¥36 000) (US $1824 [US $1688-$5091]).

^d^ The annual expense to income ratio is defined as the proportion of caregiving-related expenses incurred by the caregiver relative to the caregiver’s household income in the past year.

^e^ The HDI was proposed by the United Nations Development Programme and is a composite measure of socioeconomic development, including life expectancy, education, and gross income per capita index. The low HDI regions (HDI thresholds: ≤0.708; ≤25th percentile of HDI) include Guizhou, Qinghai Xizang, Yunnan, Anhui, Gansu, Guangxi, and Sichuan. The middle HDI regions (HDI thresholds: >0.708-0.754; >25th to 75th percentile of HDI) include Hainan, Hebei, Henan, Heilongjiang, Jiangxi, Ningxia, Shanxi, Xinjiang, Fujian, Hubei, Hunan, Jilin, Inner-Mongolia, Shandong, Shannxi, and Chongqing. The high HDI regions (HDI thresholds: >0.754; >75th percentile of HDI) include Guangdong, Jiangsu, Liaoning, Zhejiang, Beijing, Shanghai, and Tianjin.

Socioeconomic disparities were also associated with caregiving expenditures: adult child caregivers, those without cocaregivers, and those in higher SES groups reported higher absolute costs (Table 4). Strikingly, 31.7% of caregivers faced expenses exceeding their annual household income, with pronounced SES stratification (57.2% in the lowest vs 14.3% in the highest; *P* < .001). Spouse caregivers were more likely to experience financial overextension than adult child caregivers (37.8% vs 28.7%; *P* = .002). No statistically significant regional variation was observed by High Development Index (Table 4).

### Income Loss for Employed Caregivers

Among 3219 employed caregivers, 2338 (72.6%) reported work absences due to caregiving, with 845 (26.3%) losing more than 5 workdays per month and 792 (24.6%) experiencing 20% or more monthly income loss. There was a positive correlation between SES and workday loss (ρ, 0.079; *P* < .001) and monthly income loss (ρ, 0.097; *P* < .001) (eFigure 4 in the Supplement). However, caregivers with lower SES were more likely to experience severe income loss of 30% or more (23.9% in lowest SES vs 6.9% in highest SES; *P* < .001) (eTable 11 in Supplement 1).

## Discussion

This multicenter study shows the profound and multifaceted impact of socioeconomic disparities on family caregivers of older patients with cancer in China. These findings reveal that lower SES is independently associated with heavier multidimensional caregiving burden and elevated risk of depression and anxiety. These associations persisted after adjusting for regional SDI, underscoring the robustness of these findings; further analysis revealed a complex interaction between education and income on caregiver burden. Furthermore, the economic burden for caregivers was disproportionately distributed, with a considerable proportion of low-SES households experiencing caregiving-related expenses that surpassed their annual income. These results highlight the syndemic interplay of aging, cancer care demands, and socioeconomic disparities in China, offering novel insights into the challenges faced by family caregivers and informing the development of supportive policies.

These findings indicate that the association between SES and caregiver burden is multidimensional. Caregivers with lower SES consistently faced greater financial strain, health-related challenges, and insufficient social support, reflecting the direct influence of resource availability and health literacy on perceived burden. However, in the Chinese context, the association between SES and burden is not strictly linear. For instance, this study revealed that higher-educated adult child caregivers may experience increased anxiety and depression, likely due to deeper involvement in medical decision-making, multitasking across responsibilities, and adherence to filial piety norms.^21^ This contrasts with findings from high-income aging countries, where higher-educated caregivers often opt for professional services and formal care institutions.^11^ In China, even affluent individuals typically prefer personal care over formal services.^16,22^ Moreover, the concentration of patients in large public hospitals helps reduce disparities in health care access across income groups,^23,24^ yet simultaneously intensifies challenges related to time coordination and work-care balance among caregivers with high SES, which standardizes care and reduces disparities in health care access between high- and low-income groups. Therefore, understanding the link between SES and caregiver burden in China requires careful consideration of the shaping roles played by cultural values and health care system characteristics.

Further analysis revealed a complex interplay between education and income in influencing caregiver burden. While higher income alleviated financial pressure, higher education was associated with increased psychological burden, potentially through greater caregiving engagement and elevated self-expectations. This may be explained by the fact that highly educated caregivers, often with better health literacy, tend to participate more intensively in medical decisions and daily management.^25^ While this cognitive involvement can improve care quality, it may also lead to higher anxiety and self-imposed demands. On the other hand, caregivers with low education and low income face tangible economic and skill-related difficulties; yet, within high-income groups, lower education was associated with reduced psychological risk. This suggests that economic resources may buffer some stresses associated with low education and underscores fundamental differences in stressors across SES groups. These findings emphasize that SES should not be examined through a single dimension; a comprehensive assessment of the combined and interacting effects of education and income is essential to fully understand caregiver burden.

Patients with poorer health and malnutrition risk were associated with substantially increased caregiver burden, aligning with previous research.^26,27^ Female spouse caregivers assumed a higher caregiving burden, consistent with findings from previous studies.^28,29,30^ Nevertheless, male adult child caregivers appear to experience higher burden, particularly facing difficulties in scheduling and lack of family support, which indicates that gender roles and societal expectations might meaningfully influence the allocation of caregiving duties within the family unit, potentially presenting unique challenges for male caregivers. Caregivers with health problems, insufficient sleep, lack of cocaregivers, and prolonged caregiving report heavier burdens, with spouse caregivers being particularly vulnerable. Better nursing skills mitigated burden across multiple domains, whereas employed adult child caregivers experienced higher burden than those not employed. These insights can aid in identifying high-risk caregivers and guide targeted support, such as respite care and nursing-skill training, to alleviate caregiving strain.

A published review reported that caregivers in high-income countries incur direct costs ranging from CAD $300 to $14 676 (US $213-$10 441) annually.^31^ In contrast, this study revealed a median annual direct cost of ¥44 980 (CAD $8681) among Chinese caregivers. These disparities arise not only from differences in cost composition and cultural norms, but also from variations in health care financing systems. Many high-income countries provide universal health coverage and safety-net programs. For instance, in the US, Medicaid serves as the largest payer for home and community-based services, enabling care recipients with low income to access subsidized long-term care after spending down income and assets.^32,33^ This subsidization expands coverage and service use among older individuals with low income, alleviating caregivers’ strain and considerably improving their mental health.^34,35^ In China, basic medical insurance schemes, including Urban Employee Basic Medical Insurance, Urban-Rural Resident Basic Medical Insurance, and the New Rural Cooperative Medical Scheme, offer baseline coverage but still entail substantial out-of-pocket payments for advanced treatments, targeted medications, and rehabilitative care. Notably, caregivers in the present study shouldered more than 57% of patients’ medical and care-related costs, reflecting gaps in insurance coverage. The economic burden further diverges across socioeconomic strata. We introduced the expense to income ratio to contextualize caregiving costs relative to household financial capacity—a critical metric given China’s pronounced income inequality. For instance, Urban Employee Basic Medical Insurance enrollees typically experience higher reimbursement rates than Urban-Rural Resident Basic Medical Insurance/New Rural Cooperative Medical Scheme participants, yet coverage disparities persist even within these groups. Beyond direct costs, long-term caregiving imposes employment-related burdens: more than 70% of employed caregivers reported disruptions to regular work hours, disproportionately affecting individuals with higher SES who often face rigid professional expectations. This underscores the urgent need for policy interventions, including expanded insurance coverage for excluded services, income-based subsidies, and workplace accommodations (such as flexible schedules and paid caregiving leave), to mitigate financial strain and preserve caregivers’ economic stability.

This study highlights the urgent need to address socioeconomic disparities in caregiving burden through systemic reforms tailored to China’s rapidly aging population. This challenge is also highly relevant to other developing countries undergoing similar demographic transitions. Unlike high-income aging nations, China confronts health care and socioeconomic disparities.^36,37^ The rapid demographic shift has led to a substantial decrease in the number of living children per older person, indicating a decline in the caregiver-to-older person ratio. This demographic trend poses substantial challenges for both family-based and societal care systems.^16^ These disparities not only threaten caregivers’ well-being, but also compromise the quality of life and clinical outcomes of older patients with cancer, as evidenced by prior studies linking caregiver distress to delayed treatment adherence and poorer survival rates.^38,39^ To mitigate these issues, context-specific care models must be developed, including an integrated long-term care system combining home-, community-, and medical-based services. Scaling community care networks could reduce family reliance.^22^ Targeted financial protections—such as subsidies covering 30% to 50% of care-related expenses for families with low income—are imperative to alleviate catastrophic spending. Concurrently, legislative mandates for paid caregiving leave and flexible work arrangements could address the disproportionate income loss among employed caregivers. Tailored support policies must account for caregivers’ socioeconomic backgrounds and practical needs, requiring coordinated efforts across government, institutions, and society.

### Limitations

This study has several limitations. Although geographical and demographic factors were taken into account in selecting hospitals, the sample predominantly included large general and specialized hospitals, excluding subsecondary and community hospitals. Additionally, the cross-sectional design limits insights into how caregiver burden evolves over time. Third, while factors such as cancer type and stage were included as covariates, specific subgroups such as end-of-life caregivers were not separately analyzed, which may have introduced variability in reported caregiver burden. Fourth, household income was not adjusted for family size, which may have affected the precision of SES indicators. Fifth, voluntary participation may have introduced selection bias. Addressing these limitations in subsequent research will enhance the comprehension of caregiver burden across a range of settings.

## Conclusions

This cross-sectional study reveals considerable socioeconomic disparities in caregiving burden for families of older patients with cancer in China. Caregivers at the lowest SES levels experienced the most intense caregiving-related burden, elevating psychological distress and heavier economic burden, whereas higher socioeconomic groups faced employment-related costs due to caregiving-induced income loss. These inequities highlight the necessity of targeted financial support for vulnerable families, workplace protections for caregivers, and strengthened community-based care systems. Such policy measures align with the Chinese aging population strategy and offer actionable insights for low- and middle-income countries facing similar demographic transitions. Subsequent research should evaluate the cost-effectiveness of integrating caregiver support into universal health coverage frameworks.

## Notes

Supplement 1.
eMethods

eReferences
**eFigure 1.** The geographical distributions of selected hospitals and included cancer patients covered in each province **eFigure 2.** Flowchart of the study population **eFigure 3.** The proportions of expense for cancer patients provided by caregivers **eFigure 4.** The distributions of the loss of work days and income per month due to caregiving responsibilities by SES scores among employed caregivers displayed by the boxplot **eTable 1.** The list of selected hospitals **eTable 2. **Provincial-level Social Deprivation Index Values in China **eTable 3.** The mean and standard deviation scores for five subscales of Caregiver Reaction Assessment (CRA) scale by SES quartiles **eTable 4.** The generalized linear model for factors associated with total scores of each subscale of CRA for all, spouse, and adult-child caregivers **eTable 5.** The sensitivity analysis further adjusting for the Social Deprivation Index (SDI) in the generalized linear models **eTable 6.** Associations of educational level and household income level with the score of CRA-subscale stratified by household income level or educational level **eTable 7.** The proportions of caregivers with the anxiety and depression symptom by SES quartiles **eTable 8. **The associations between SES status and depression anxiety and symptom in the sensitivity analysis further adjusting for the SDI **eTable 9.** Association of household income level or educational level with the risk of depression and anxiety symptom stratified by educational level or household income level **eTable 10.** The direct caregiving-related expense for cancer patients in the past year **eTable 11.** The indirect economic burden for employed caregivers by SES quartiles

Supplement 2.
Data Sharing Statement
