# Supplementary material for: Socioeconomic Disparities in Caregiver Burden Among Families of Older Patients With Cancer
Source: JAMA Health Forum. 2025 Dec 26;6(12):e255614. doi: 10.1001/jamahealthforum.2025.5614 (PMC12743282; doi:10.1001/jamahealthforum.2025.5614)
Supplement: Supplement 1. — eMethods eReferences eFigure 1. The geographical distributions of selected hospitals and included cancer patients covered in each province eFigure 2. Flowchart of the study population eFigure 3. The proportions of expense for cancer patients provided by caregivers eFigure 4. The distributions of the loss of work days and income per month due to caregiving responsibilities by SES scores among employed caregivers displayed by the boxplot eTable 1. The list of selected hospitals eTable 2. Provincial-level Social Deprivation Index Values in China eTable 3. The mean and standard deviation scores for five subscales of Caregiver Reaction Assessment (CRA) scale by SES quartiles eTable 4. The generalized linear model for factors associated with total scores of each subscale of CRA for all, spouse, and adult-child caregivers eTable 5. The sensitivity analysis further adjusting for the Social Deprivation Index (SDI) in the generalized linear models eTable 6. Associations of educational level and household income level with the score of CRA-subscale stratified by household income level or educational level eTable 7. The proportions of caregivers with the anxiety and depression symptom by SES quartiles eTable 8. The associations between SES status and depression anxiety and symptom in the sensitivity analysis further adjusting for the SDI eTable 9. Association of household income level or educational level with the risk of depression and anxiety symptom stratified by educational level or household income level eTable 10. The direct caregiving-related expense for cancer patients in the past year eTable 11. The indirect economic burden for employed caregivers by SES quartiles [file jamahealthforum-e255614-s001.pdf]

Supplemental Online Content

Ju W, Jiang H, Ma S, et al. Socioeconomic disparities in caregiver burden among families of older patients with cancer. *JAMA Health Forum*. 2025;6(12):e255614.
doi:10.1001/jamahealthforum.2025.5614

eMethods
eReferences
eFigure 1. The geographical distributions of selected hospitals and included cancer patients covered in each province
eFigure 2. Flowchart of the study population
eFigure 3. The proportions of expense for cancer patients provided by caregivers
eFigure 4. The distributions of the loss of work days and income per month due to caregiving responsibilities by SES scores among employed caregivers displayed by the boxplot
eTable 1. The list of selected hospitals
eTable 2. Provincial-level Social Deprivation Index Values in China
eTable 3. The mean and standard deviation scores for five subscales of Caregiver Reaction Assessment (CRA) scale by SES quartiles
eTable 4. The generalized linear model for factors associated with total scores of each subscale of CRA for all, spouse, and adult-child caregivers
eTable 5. The sensitivity analysis further adjusting for the Social Deprivation Index (SDI) in the generalized linear models
eTable 6. Associations of educational level and household income level with the score of CRA-subscale stratified by household income level or educational level
eTable 7. The proportions of caregivers with the anxiety and depression symptom by SES quartiles
eTable 8. The associations between SES status and depression anxiety and symptom in the sensitivity analysis further adjusting for the SDI
eTable 9. Association of household income level or educational level with the risk of depression and anxiety symptom stratified by educational level or household income level
eTable 10. The direct caregiving-related expense for cancer patients in the past year
eTable 11. The indirect economic burden for employed caregivers by SES quartiles

This supplemental material has been provided by the authors to give readers additional information about their work.

© 2025 Ju W et al. JAMA Health Forum.

eMethods

Collection of baseline information

The collected data for cancer patients included demographic information (age, sex, race/ethnicity, marital status, number of children, type of medical insurance), health status (EuroQol-5D-5L questionnaire (EQ-5D-5L)), nutritional status (Body mass index (BMI), grip strength, calf/arm circumference, SARC-F questionnaire, Nutritional Risk Screening 2002 (NRS2002)), and clinical information (cancer type, date of diagnosis, stage, grade, comorbidities, treatment, hospitalization costs). Information of caregivers encompassed socio-demographic details (age, sex, race/ethnicity, self-reported health problems, employment status, education, relationship with the patient, occupation, individual and household income), caregiving-related information (Co-caregiver availability, duration of current care episode (days), daily sleep hours, and self-reported nursing proficiency (extremely skilled, moderately skilled, not skilled), caregiving-related expenses (direct medical cost and direct non-medical cost), and the impact on employment (loss of work time and income).

1. Health measurements of older cancer patients

- Hand grip strength was assessed using the JAMAR hydraulic hand dynamometer (MESM) and was recorded as the mean of three trials for each hand.
- The maximal calf circumference was measured on the right calf with the participant in a seated position. A measuring tape was slid along the calf's length to identify the maximum circumference in a plane orthogonal to the calf's long axis. Calf circumference was recorded to the nearest 0.1 cm.
- Arm circumference was taken with the participant standing upright, arms relaxed at their

© 2025 Ju W et al. JAMA Health Forum.

sides. The measuring tape was wrapped around the right upper arm, ensuring it was perpendicular to the arm's long axis at the mid-point. Arm circumference was documented to the nearest millimeter.

2. EuroQol-5D-5L questionnaire

The EQ-5D-5L is an instrument used to assess health status and health-related quality of life (HRQoL)[1] for cancer patients. The descriptive system of the EQ-5D comprises five dimensions: mobility (MO), self-care (SC), usual activities (UA), pain/discomfort (PD), and anxiety/depression (AD); each dimension is described at five levels, ranging from no problems to extreme problems. In this study, we employed the EQ-5D-5L value set for China estimated by Luo et.al.[2]

3. SARC-F

The SARC-F is a simple questionnaire designed for the rapid diagnosis of sarcopenia, which is characterized by muscle loss. This questionnaire screens for signs of sarcopenia-related symptoms reported by patients, such as strength decline, difficulty walking, difficulty rising from a chair, difficulty climbing stairs, and a history of falls, to identify the risk of sarcopenia. The scores on SARC-F range from 0 to 10, with each component being scored from 0 to 2 points; a score of 4 or higher is indicative of sarcopenia and poor outcomes.[3]

4. Nutritional risk screening (NRS 2002)

The NRS 2002 is a tool developed by the European Society of Parenteral and Enteral Nutrition (ESPEN) for assessing nutritional risk. It is based on 128 randomized controlled clinical studies, making it the only screening tool that has been validated with such evidence-based research. The total score of NRS 2002 is calculated by summing the scores for disease

© 2025 Ju W et al. JAMA Health Forum.

severity, nutritional status impairment, and age (with an additional point added for individuals over 70 years old). A score of 3 or more indicates a nutritional risk.[4]

5. Socioeconomic status (SES)

We developed a composite SES score that integrated household income from the past year and educational level for caregivers. The detailed categories are outlined in the following table.

1) The definition of SES score

Household income in the past year (in survey)	SES score1	Educational background (in survey)	SES score2
<20000	1	Never received formal education	1
20000-39999	2	Elementary school	2
40000-59999	3	Junior high school	3
60000-79999	4	High school	4
80000-99999	5	Vocational college	5
100000-149999	6	Bachelor's degree	6
150000-249999	7	Master's degree or above	7
≥250000	8		

The final SES score was a composite of SES score1 and score2, spanning a range from 2 to 16.

2) The definition of educational level

© 2025 Ju W et al. JAMA Health Forum.

Educational background (in survey)	Educational level
Never received formal education<br>Elementary school<br>Junior high school	Primary
High school<br>Vocational college	Secondary
Bachelor's degree<br>Master's degree or above	Tertiary

3) The definition of household income level

Household income in the past year (in survey)	Household income level
<20000	Quartile 1
20000-39999<br>40000-59999	Quartile 2
60000-79999<br>80000-99999	Quartile 3
100000-149999<br>150000-249999<br>≥250000	Quartile 4

The SES of caregiver was categorized into four groups according to the combination of

© 2025 Ju W et al. JAMA Health Forum.

household income (1 to 4) and education level (1 to 3): the lowest SES (2 to 3), lower-middle SES (4), upper-middle SES (5), and highest SES (6 to 8).

6. The Provincial-level Social Deprivation Index (SDI) Values in China

The provincial-level Social Deprivation Index used in this analysis was sourced from the study by Zhu et al.[5] This composite measure was constructed using the Coefficient of Variation method based on multidimensional data from Chinese 2020 Seventh National Population Census, encompassing key domains such as income, employment, education, housing conditions, and demographic structure. Higher values of the SDI indicate greater levels of social deprivation within a province. Detailed values are provided in eTable2.

7. Caregiver Reaction Assessment (CRA) scale

The CRA scale was used to assess the burden experienced by caregivers, comprising 24 items that elicit both positive and negative responses to caregiving.[6] This multidimensional measure employs a 5-point scale, where 1 indicates strong agreement and 5 indicates strong disagreement. The items are grouped into five subscales: health, self-esteem, schedule, finance and family support.

- Health: the subscale of health measures the caregiver’s physical capability and energy to provide care. It also assesses the caregiver’s health in relation to the caregiving role.
- Self-esteem: the subscale of caregiver’s self-esteem measures the extent to which caregiving imparts individual self-esteem. Items assess whether caregiving is enjoyable and rewarding or whether it causes resentment.
- Schedule: the subscale of schedule measures the extent to which caregiving interrupts usual activities, causes the elimination of some activities, and interferes with relaxation time.

© 2025 Ju W et al. JAMA Health Forum.

- Finance: the subscale of finance measures the adequacy, the difficulty, and the strain of the financial situation on the caregiver and the family.
- Family support: the subscale of family support measures the extent to which family supports and works together with the caregiver.

For each subscale, the average score was calculated, allowing subscale scores to potentially range from 1 to 5. To enhance interpretability and ensure consistency across all subscales, the self-esteem item was reverse-scored so that higher values uniformly indicate a greater burden. Thus, for all five subscales: health, self-esteem, schedule, finance, and lack of family support, a higher score reflects a higher level of caregiver burden. The CRA has been validated in various clinical settings and cultural contexts.[7-11]

8. Patient Health Questionnaire 9 (PHQ-9)

The depressive symptoms of caregivers were assessed using the PHQ-9, which is based on the diagnostic criteria from the Diagnostic and Statistical Manual of Mental Disorders, Fifth Edition (DSM-5). This scale consists of nine items, each rated on a scale from 0 to 3, with higher total scores reflecting greater depressive symptom. The scores span a range from 0 to 4, indicating no depression, 5 to 9 indicating the presence of depressive symptoms, 10 to 14 suggesting significant depressive symptoms, 15 to 19 denoting moderate to severe depression, and scores above 20 indicating severe depression. The reliability and validity of the Chinese version of the scale have been established in prior research.[12,13]

9. Generalized Anxiety Disorder scale 7 (GAD-7)

The GAD-7 scale is a commonly used instrument for evaluating the anxiety symptom, widely employed in clinical settings and as a preliminary screening tool within primary

© 2025 Ju W et al. JAMA Health Forum.

healthcare. The scale encompasses three domains: physical functions, cognitive aspects, and emotional experiences. Similar to the PHQ-9, the GAD-7 score correlates positively with the severity of anxiety, with the following thresholds: 0–4 indicating no anxiety, 5–9 suggesting mild anxiety, 10–14 indicating moderate anxiety, and 15–21 denoting severe anxiety. The reliability and validity of the Chinese version of the GAD-7 scale have been established in previous studies.[14,15]

10. The economic burden for the family caregiver

We assessed direct caregiving costs using data obtained from structured face-to-face interviews. Direct costs were defined as expenses incurred by family caregivers when accompanying patients to hospital-based treatments over the previous year. These costs included:

- Direct medical expenses: patient treatment costs.
- Direct non-medical expenses: transportation, hospital lodging, nutritional supplements, and nursing services, calculated annually.

We also calculated the annual expense-to-income ratio, which is the proportion of caregiving-related expenses relative to the caregiver's household income for the same period, to quantify financial burden relative to caregivers' total household income.

Additionally, employment-related costs were defined as income losses resulting from caregivers' work absences, late arrivals, or early departures necessitated by medical appointments or home care duties. Employed caregivers reported days absent and income lost due to caregiving in the preceding month, including forfeited full-attendance bonuses. Monthly worktime and income losses were stratified by socioeconomic status (SES) groups, and

© 2025 Ju W et al. JAMA Health Forum.

associations between workday loss, income loss, and SES scores were analyzed.

eReferences

1. Health-related quality of life (HRQoL). http://www.euroqol.org

2. Luo N, Liu G, Li M, Guan H, Jin X, Rand-Hendriksen K. Estimating an EQ-5D-5L Value Set for China. *Value Health*. Apr 2017;20(4):662-669. doi:10.1016/j.jval.2016.11.016

3. Malmstrom TK, Morley JE. SARC-F: a simple questionnaire to rapidly diagnose sarcopenia. *J Am Med Dir Assoc*. Aug 2013;14(8):531-2. doi:10.1016/j.jamda.2013.05.018

4. Kondrup J, Rasmussen HH, Hamberg O, Stanga Z, Ad Hoc EWG. Nutritional risk screening (NRS 2002): a new method based on an analysis of controlled clinical trials. *Clin Nutr*. Jun 2003;22(3):321-36. doi:10.1016/s0261-5614(02)00214-5

5. Zhu Y, Ding Y, Qin S. Investigating the relationship between social deprivation and health outcomes in China: using spatial regression analysis approach. *BMC Public Health*. Mar 11 2025;25(1):951. doi:10.1186/s12889-025-22140-5

6. Given CW, Given B, Stommel M, Collins C, King S, Franklin S. The caregiver reaction assessment (CRA) for caregivers to persons with chronic physical and mental impairments. *Res Nurs Health*. Aug 1992;15(4):271-83. doi:10.1002/nur.4770150406

7. Ge C, Yang X, Fu J, et al. Reliability and validity of the Chinese version of the Caregiver Reaction Assessment. *Psychiatry Clin Neurosci*. Apr 2011;65(3):254-63. doi:10.1111/j.1440-1819.2011.02200.x

8. Alvira C, Cabrera E, Kostov B, et al. Validity and reliability of the Spanish caregiver reaction assessment scale for caregivers of people with dementia. *Int J Nurs Pract*. Aug 2021;27(4):e12848. doi:10.1111/ijn.12848

© 2025 Ju W et al. JAMA Health Forum.

9. Malhotra R, Chan A, Malhotra C, Ostbye T. Validity and reliability of the Caregiver Reaction Assessment scale among primary informal caregivers for older persons in Singapore. *Aging Ment Health*. 2012;16(8):1004-15. doi:10.1080/13607863.2012.702728

10. Misawa T, Miyashita M, Kawa M, et al. Validity and reliability of the Japanese version of the Caregiver Reaction Assessment Scale (CRA-J) for community-dwelling cancer patients. *Am J Hosp Palliat Care*. Oct-Nov 2009;26(5):334-40. doi:10.1177/1049909109338480

11. Yang HK, Shin DW, Kim SY, et al. Validity and reliability of the Korean version of the caregiver reaction assessment scale in family caregivers of cancer patients. *Psychooncology*. Dec 2013;22(12):2864-8. doi:10.1002/pon.3364

12. Ye X, Shu HL, Feng X, et al. Reliability and validity of the Chinese version of the Patient Health Questionnaire-9 (C-PHQ-9) in patients with psoriasis: a cross-sectional study. *BMJ Open*. Jul 13 2020;10(7):e033211. doi:10.1136/bmjopen-2019-033211

13. Sun Y, Kong Z, Song Y, Liu J, Wang X. The validity and reliability of the PHQ-9 on screening of depression in neurology: a cross sectional study. *BMC Psychiatry*. Feb 9 2022;22(1):98. doi:10.1186/s12888-021-03661-w

14. Sun J, Liang K, Chi X, Chen S. Psychometric Properties of the Generalized Anxiety Disorder Scale-7 Item (GAD-7) in a Large Sample of Chinese Adolescents. *Healthcare (Basel)*. Dec 9 2021;9(12)doi:10.3390/healthcare9121709

15. Zhang C, Wang T, Zeng P, et al. Reliability, Validity, and Measurement Invariance of the General Anxiety Disorder Scale Among Chinese Medical University Students. *Front Psychiatry*. 2021;12:648755. doi:10.3389/fpsyt.2021.648755

© 2025 Ju W et al. JAMA Health Forum.

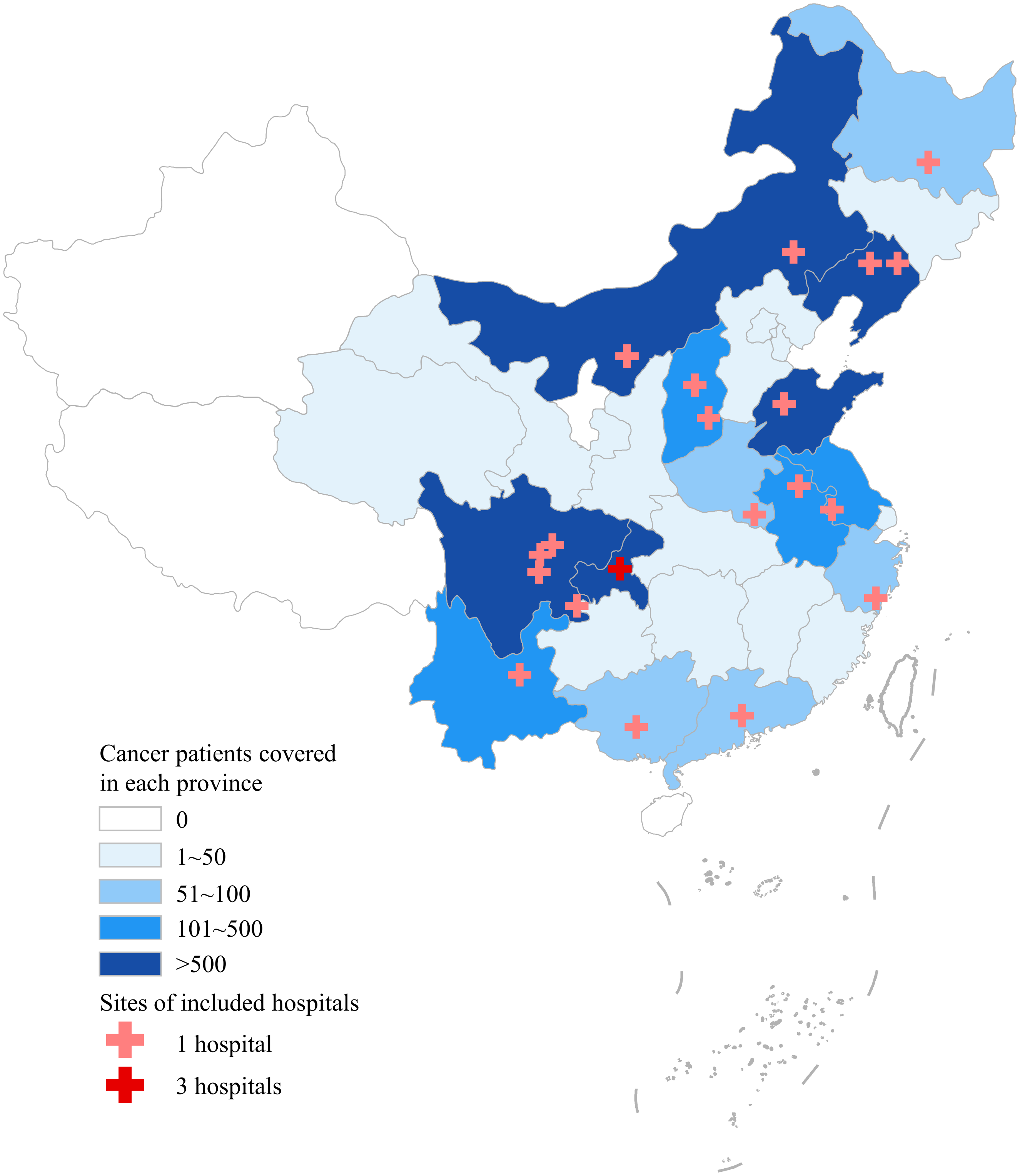

eFigure 1. The geographical distributions of selected hospitals and included cancer patients covered in each province.

© 2025 Ju W et al. JAMA Health Forum.

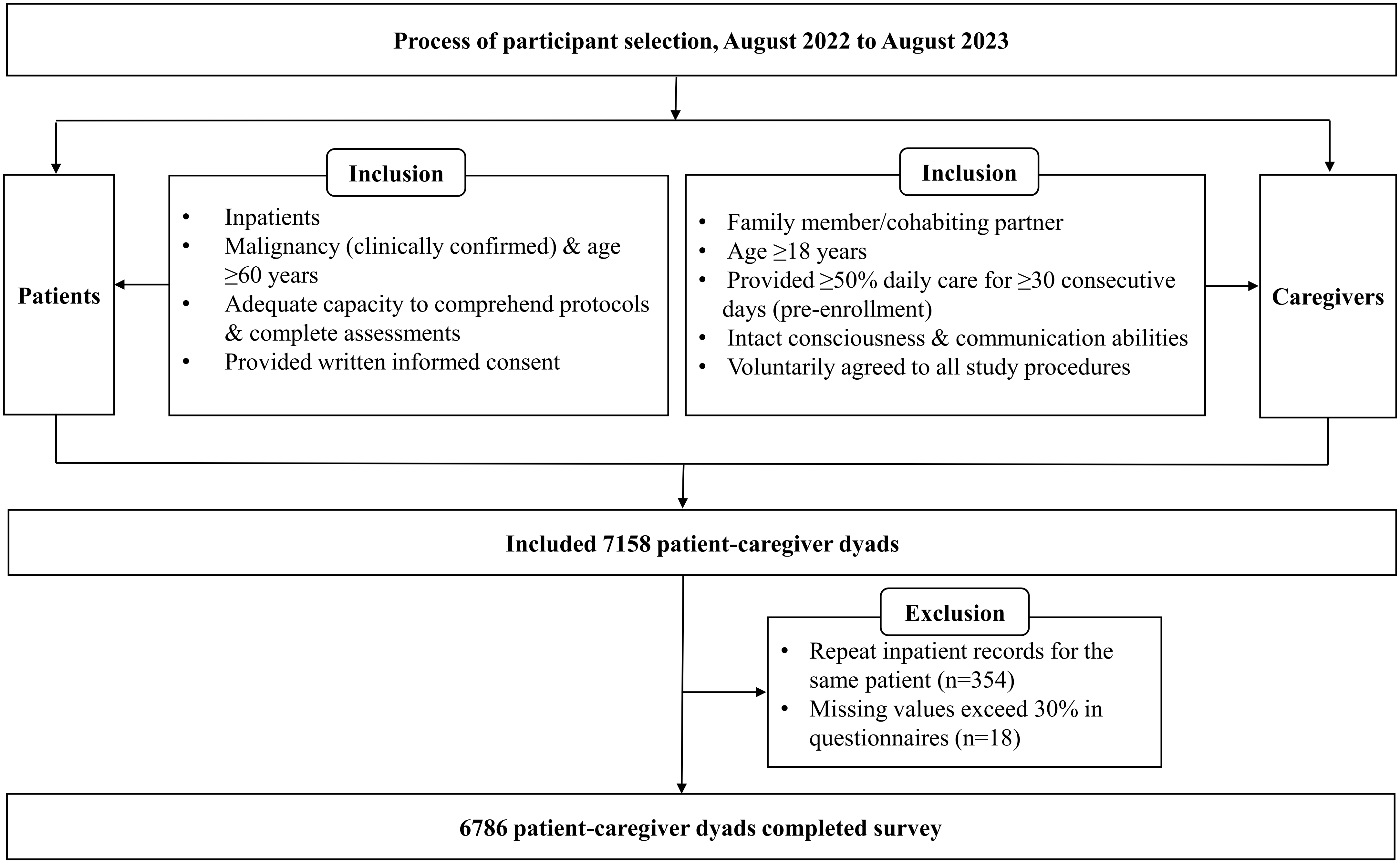

© 2025 Ju W et al. JAMA Health Forum.

eFigure 2. Flowchart of the study population.

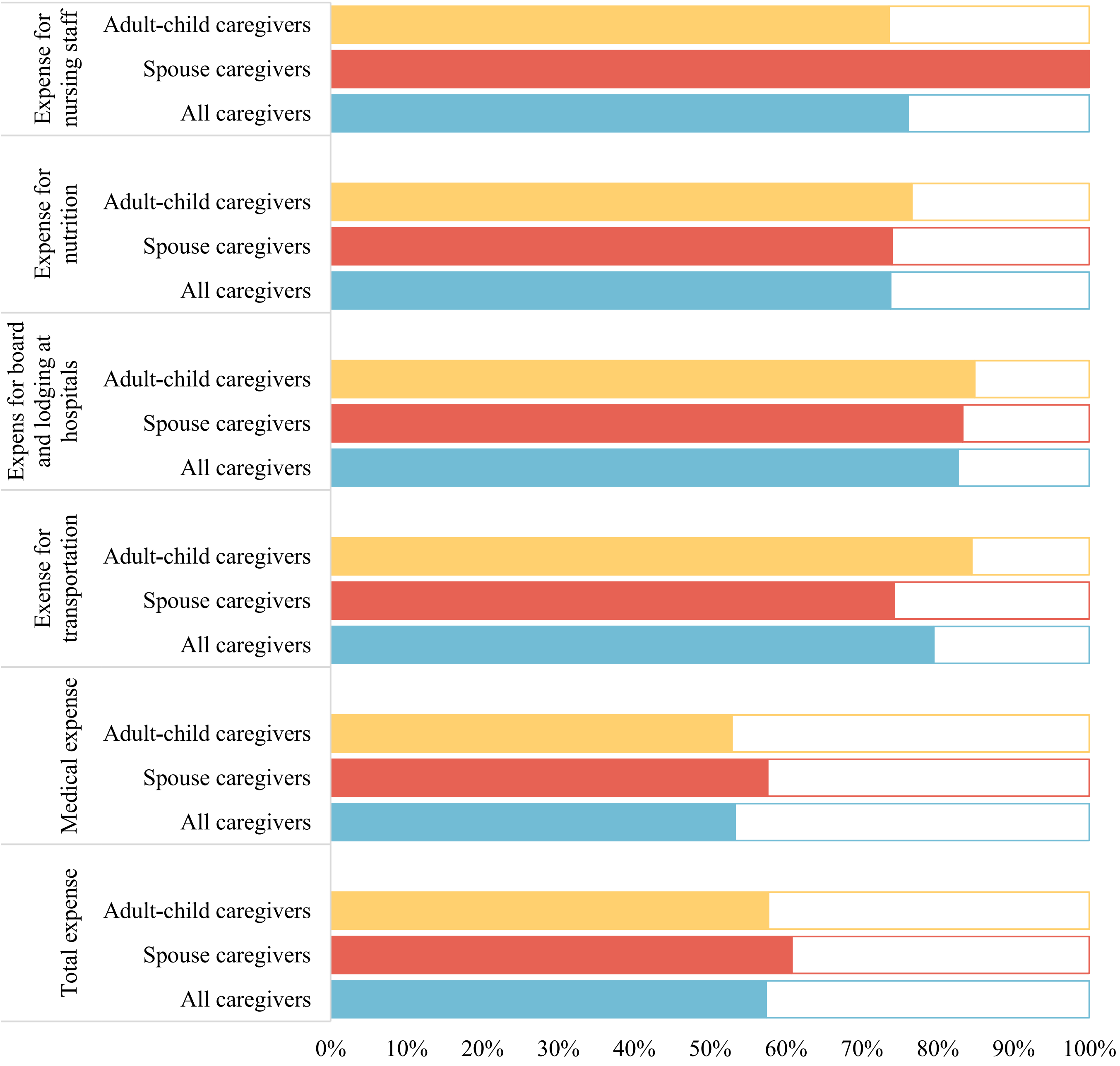

eFigure 3. The proportions of expense for cancer patients provided by caregivers.

© 2025 Ju W et al. JAMA Health Forum.

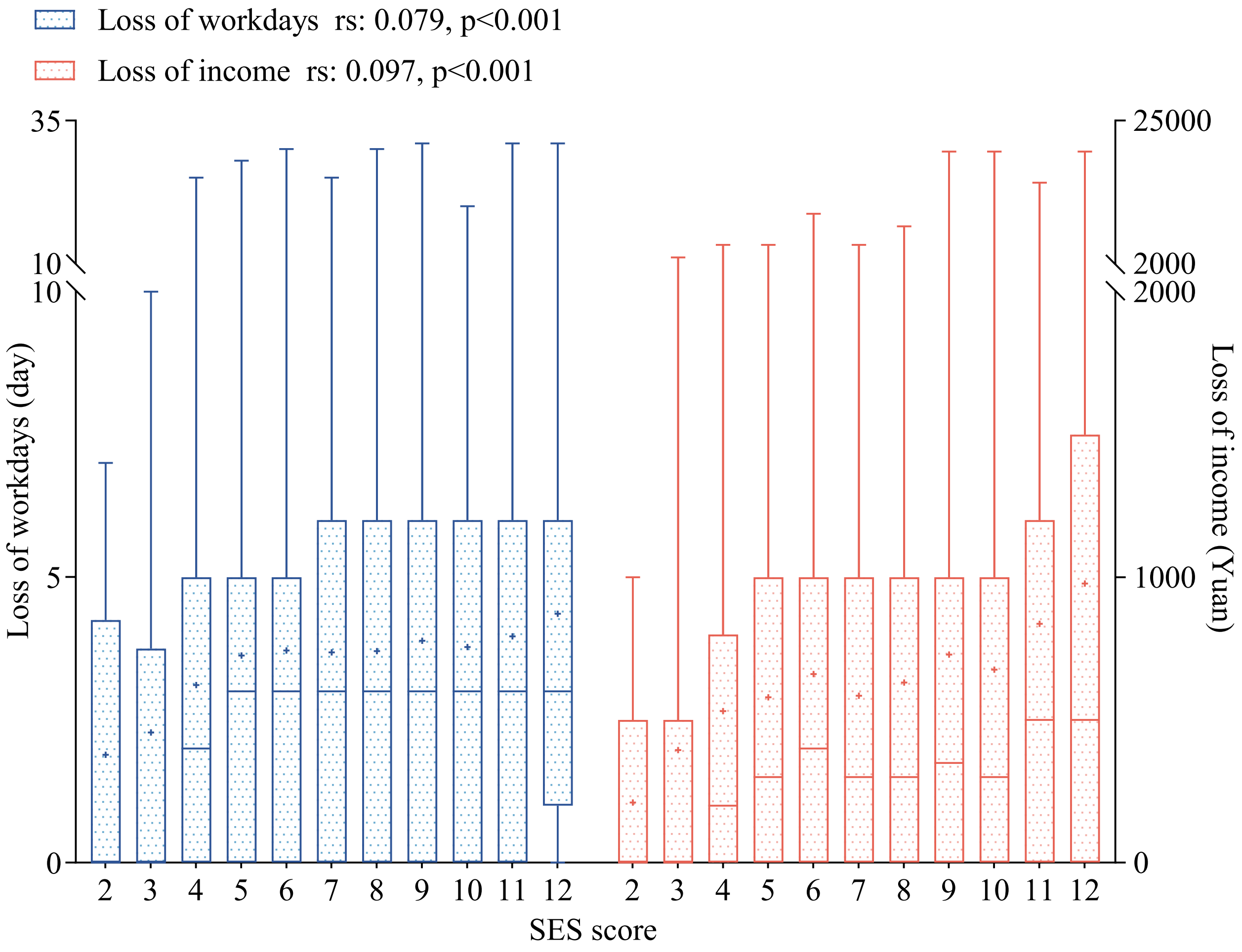

eFigure 4. The distributions of the loss of work days and income per month due to caregiving responsibilities by SES scores among employed caregivers displayed by the boxplot.

r_s was the Spearman rank correlation coefficient of the relation between SES scores and loss of work days (loss of income); p-values <0.05 suggests a statistically significant rank correlation.

© 2025 Ju W et al. JAMA Health Forum.

eTable 1 The list of selected hospitals

Province	Selected Hospital
**Eastern Region**	
Guangdong	Southern Hospital of Southern Medical University
Jiangsu	Jiangsu Province Hospital
Liaoning	Liaoning Cancer Hospital
	The People's Hospital of Liaoning Province
Shandong	Shandong Cancer Hospital
Zhejiang	The First Affiliated Hospital of Wenzhou Medical College
**Central Region**	
Anhui	The First Affiliated Hospital of Bengbu Medical College
Henan	Xinyang Central Hospital
Heilongjiang	The Affiliated Cancer Hospital of Harbin Medical University
Shanxi	Bai Qiu'en (Bethune) Hospital of Shanxi Province
	Changzhi People's Hospital of Shanxi Province
**Western Region**	
Inner-Mongolia	Chifeng City Hospital
	Ordos Central Hospital
Sichuan	Deyang Cancer Hospital
	Dujiangyan City Hospital
	Sichuan Meishan Traditional Chinese Medicine Hospital
	Sichuan Meishan Cancer Hospital
Yunnan	Yunnan Cancer Hospital
Chongqing	The Affiliated Cancer Hospital of Chongqing University
	The Second People's Hospital of Chongqing Jiangjin District
	Chongqing Qijiang District People's Hospital
Guangxi	The First Affiliated Hospital of Guangxi Medical University

© 2025 Ju W et al. JAMA Health Forum.

eTable 2 Provincial-level Social Deprivation Index Values in China

Province	Social Deprivation Index[a]
Anhui	0.262
Beijing	0.179
Chongqing	0.266
Fujian	0.247
Gansu	0.406
Guangdong	0.228
Guangxi	0.271
Guizhou	0.363
Hainan	0.318
Hebei	0.234
Heilongjiang	0.347
Henan	0.225
Hubei	0.225
Hunan	0.244
Inner Mongolia	0.418
Jiangsu	0.18
Jiangxi	0.206
Jilin	0.311
Liaoning	0.36
Ningxia	0.238
Qinghai	0.459
Shaanxi	0.289
Shandong	0.235
Shanghai	0.197
Shanxi	0.364
Sichuan	0.343
Tianjin	0.181
Tibet	0.882
Xinjiang	0.227
Yunnan	0.368
Zhejiang	0.241

[a] Social Deprivation Index calculated via the Coefficient of Variation Method; Higher values indicate a higher degree of social deprivation.

© 2025 Ju W et al. JAMA Health Forum.

eTable 3 The mean and standard deviation scores for five subscales of Caregiver Reaction Assessment (CRA) scale by SES quartiles.

**CRA subscales**	Overall	**SES** quartiles				P-value
		Lowest	Lower-middle	Upper-middle	Highest	
All caregivers						
N	6609	1866	1727	1313	1703	
Heath	2.02±0.69	2.18±0.68	2.02±0.68	1.97±0.69	1.88±0.67	<0.001
Self-esteem	2.39±0.42	2.45±0.42	2.41±0.39	2.38±0.42	2.29±0.42	<0.001
Scheduling	2.64±0.72	2.73±0.6	2.6±0.72	2.59±0.73	2.62±0.81	0.032
Finance	3.03±0.74	3.12±0.69	3.18±0.65	3.04±0.74	2.79±0.81	<0.001
Family support	2.09±0.56	2.24±0.5	2.08±0.53	2.02±0.56	2.01±0.6	<0.001
Spouse caregivers						
N	2801	1125	789	493	394	
Heath	2.22±0.73*	2.30±0.69	2.18±0.76	2.22±0.77	2.07±0.73	<0.001
Self-esteem	2.45±0.35	2.48±0.35	2.44±0.34	2.43±0.36	2.43±0.38	0.001
Scheduling	2.62±0.72	2.77±0.59	2.55±0.77	2.54±0.78	2.45±0.81	<0.001
Finance	3.15±0.69*	3.17±0.68	3.22±0.62	3.14±0.73	2.97±0.75	0.024
Family support	2.12±0.54*	2.24±0.49	2.07±0.54	2.02±0.59	1.99±0.54	<0.001
Adult-child caregivers						
N	3372	641	827	717	1187	
Heath	1.88±0.62	2.01±0.63	1.89±0.58	1.82±0.6	1.83±0.64	<0.001
Self-esteem	2.32±0.45†	2.38±0.50	2.37±0.42	2.33±0.46	2.24±0.42	<0.001
Scheduling	2.70±0.69†	2.69±0.58	2.69±0.64	2.66±0.65	2.71±0.8	0.008
Finance	2.96±0.76	3.06±0.70	3.14±0.67	2.98±0.74	2.75±0.81	<0.001
Family support	2.08±0.56	2.23±0.50	2.08±0.52	2.02±0.54	2.03±0.62	<0.001

*The mean score of the subscale among spouse caregivers is significantly higher than that of adult-child caregivers (p<0.001). †The mean score of the subscale among adult-child caregivers is significantly higher than that of spouse caregivers (p<0.001).

© 2025 Ju W et al. JAMA Health Forum.

eTable 4A The generalized linear model for factors associated with total scores of each subscale of CRA for all caregivers.

	Heath		Self-esteem		Scheduling		Finance		Family support	
	Coefficient (95% CI)	p-value	Coefficient (95% CI)	p-value	Coefficient (95% CI)	p-value	Coefficient (95% CI)	p-value	Coefficient (95% CI)	p-value
Intercept	8.39 (7.78 to 8.99)	<0.001	18.59 (17.94 to 19.24)	<0.001	13.95 (13.12 to 14.79)	<0.001	9.94 (9.38 to 10.49)	<0.001	11.85 (11.26 to 122.45)	<0.001
SES Score	-0.07 (-0.10 to -0.04)	<0.001	-0.15 (-0.18 to -0.12)	<0.001	-0.07 (-0.11 to -0.03)	0.001	-0.14 (-0.17 to -0.11)	<0.001	-0.14 (-0.17 to -0.11)	<0.001
Sex (vs Male)										
Female	0.16 (0.02 to 0.30)	<0.001	-0.15 (-0.3 to 0)	0.049	-0.28 (-0.47 to -0.09)	0.004	0.01 (-0.11 to 0.14)	0.845	-0.11 (-0.24 to 0.03)	0.138
Age group (vs <60)										
≥60	0.70 (0.51 to 0.89)	<0.001	0.52 (0.31 to 0.72)	<0.001	-0.57 (-0.83 to -0.31)	<0.001	0.10 (-0.07 to 0.28)	0.264	-0.12 (-0.31 to 0.07)	0.216
Self-reported health problem (vs No)										
Yes	0.6 (0.38 to 0.82)	<0.001	-0.5 (-0.71 to -0.28)	<0.001	0.78 (0.50 to 1.07)	<0.001	-0.12 (-0.30 to 0.07)	0.220	0.01 (-0.19 to 0.21)	0.94
Sleep hours per day for caregiver (>8h)										
<6h	1.58 (1.30 to 1.85)	<0.001	0.14 (-0.17 to 0.44)	0.381	1.19 (0.79 to 1.59)	<0.001	0.84 (0.60 to 1.08)	<0.001	1.15 (0.87 to 1.43)	<0.001
6-8h	0.15 (-0.08 to 0.39)	0.209	-0.32 (-0.59 to -0.05)	0.022	-0.13 (-0.48 to 0.22)	0.465	0.94 (0.73 to 1.15)	<0.001	0.13 (-0.11 to 0.37)	0.292
Co-caregivers (vs No)										
Yes	-0.34 (-0.49 to -0.20)	<0.001	-0.59 (-0.75 to -0.43)	<0.001	-1.72 (-1.93 to -1.52)	<0.001	-0.02 (-0.15 to 0.12)	0.803	-0.84 (-0.98 to -0.69)	<0.001
Duration of Current Care Episode (days) (vs >10)										
<3	-0.93 (-1.18 to -0.68)	<0.001	-0.62 (-0.89 to -0.36)	<0.001	-1.74 (-2.06 to -1.41)	<0.001	-0.41 (-0.63 to -0.18)	<0.001	-0.63 (-0.88 to -0.39)	<0.001
3-5	-0.46 (-0.67 to -0.25)	<0.001	-0.43 (-0.66 to -0.21)	<0.001	-0.60 (-0.90 to -0.30)	<0.001	-0.43 (-0.62 to -0.23)	<0.001	-0.26 (-0.47 to -0.04)	0.018
6-9	-0.24 (-0.46 to -0.03)	0.03	-0.52 (-0.75 to -0.29)	<0.001	-0.04 (-0.34 to 0.27)	0.813	-0.28 (-0.47 to -0.08)	0.006	-0.44 (-0.65 to -0.23)	<0.001
Work status (vs Employed)										
Unemployed	0.02 (-0.17 to 0.21)	0.820	-0.06 (-0.27 to 0.14)	0.557	-1.17 (-1.45 to -0.90)	<0.001	0.23 (0.05 to 0.411)	0.011	-0.64 (-0.85 to -0.45)	<0.001

© 2025 Ju W et al. JAMA Health Forum.

Nursing care skills (vs Extremely and Moderately skilled)										
Not skilled at all	-0.004 (-0.15 to 0.14)	0.961	-0.81 (-0.96 to -0.65)	<0.001	0.68 (0.48 to 0.88)	<0.001	0.04 (-0.09 to 0.17)	0.540	-0.62 (-0.76 to -0.47)	<0.001
Prevalence year (vs ≥5)										
<1	0.28 (0.05 to 0.52)	0.016	0.15 (-0.11 to 0.4)	0.264	1.04 (0.73 to 1.35)	<0.001	0.34 (0.12 to 0.56)	0.002	0.14 (-0.10 to 0.37)	0.260
1-2.9	0.15 (-0.10 to 0.40)	0.238	0.21 (-0.06 to 0.48)	0.129	0.61 (0.29 to 0.93)	<0.001	0.15 (-0.08 to 0.38)	0.202	-0.13 (-0.38 to 0.122)	0.325
3-4.9	0.20 (-0.11 to 0.50)	0.204	-0.01 (-0.34 to 0.32)	0.956	0.42 (0.02 to 0.82)	0.039	-0.01 (-0.29 to 0.27)	0.935	-0.19 (-0.50 to 0.11)	0.21
Stage (vs IV)										
0	-0.45 (-1.01 to 0.11)	0.116	0.42 (-0.24 to 1.09)	0.214	-0.46 (-1.31 to 0.39)	0.289	-0.22 (-0.78 to 0.34)	0.438	0.07 (-0.55 to 0.69)	0.826
I	-0.23 (-0.54 to 0.07)	0.130	0.47 (0.11 to 0.82)	0.01	-0.06 (-0.50 to 0.38)	0.782	-0.34 (-0.63 to -0.05)	0.021	0.24 (-0.09 to 0.57)	0.151
II	-0.12 (-0.35 to 0.11)	0.317	0.12 (-0.14 to 0.37)	0.378	-0.02 (-0.35 to 0.30)	0.888	-0.39 (-0.60 to -0.18)	<0.001	0.24 (-0.007 to 0.48)	0.057
III	-0.08 (-0.24 to 0.08)	0.325	-0.05 (-0.22 to 0.12)	0.562	0.25 (0.02 to 0.48)	0.062	-0.01 (-0.29 to 0.27)	0.935	-0.07 (-0.23 to 0.09)	0.415
Surgery (vs No)										
Yes	-1.23 (-1.49 to -0.97)	<0.001	-0.36 (-0.68 to -0.04)	0.025	-1.23 (-1.63 to -0.83)	<0.001	-0.15 (-0.41 to 0.122)	0.288	-1.40 (-1.67 to -1.13)	<0.001
NRS2002 (vs <3)										
≥3	0.22 (0.07 to 0.37)	0.004	-0.04 (-0.2 to 0.12)	0.628	0.92 (0.71 to 1.13)	<0.001	-0.08 (-0.21 to 0.06)	0.269	0.02 (-0.13 to 0.16)	0.834
EQ-5D-5L value	-0.38 (-0.73 to -0.04)	0.031	0.46 (0.11 to 0.8)	0.009	0.10 (-0.36 to 0.55)	0.675	-0.73 (-1.05 to -0.41)	<0.001	0.84 (0.52 to 1.15)	<0.001

© 2025 Ju W et al. JAMA Health Forum.

eTable 4B The generalized linear model for factors associated with total scores of each subscale of CRA for spouse caregivers.

	Heath		Self-esteem		Scheduling		Finance		Family support	
	Coefficient (95% CI)	p-value	Coefficient (95% CI)	p-value	Coefficient (95% CI)	p-value	Coefficient (95% CI)	p-value	Coefficient (95% CI)	p-value
Intercept	10.33 (9.03 to 11.64)	<0.001	18.42 (17.34 to 19.49)	<0.001	15.76 (14.26 to 17.26)	<0.001	9.23 (8.23 to 10.24)	<0.001	12.42 (11.31 to 13.52)	<0.001
SES Score	-0.05 (-0.09 to 0)	0.044	-0.1 (-0.14 to -0.06)	<0.001	-0.17 (-0.22 to -0.11)	<0.001	-0.1 (-0.14 to -0.06)	<0.001	-0.14 (-0.18 to -0.1)	<0.001
Sex (vs Male)										
Female	0.42 (0.2 to 0.65)	<0.001	0.05 (-0.15 to 0.25)	0.639	0.18 (-0.09 to 0.45)	0.198	0.11 (-0.08 to 0.3)	0.24	0.18 (-0.03 to 0.38)	0.096
Age group (vs <60)										
≥60	-0.09 (-0.45 to 0.28)	0.639	-0.17 (-0.49 to 0.15)	<0.001	-0.53 (-0.99 to -0.07)	0.023	-0.14 (-0.44 to 0.16)	0.369	-0.35 (-0.69 to -0.02)	0.04
Self-reported health problem (vs No)										
Yes	0.5 (0.23 to 0.76)	<0.001	-0.43 (-0.65 to -0.21)	<0.001	0.54 (0.22 to 0.86)	0.001	-0.05 (-0.26 to 0.16)	0.648	0.01 (-0.22 to 0.24)	0.935
Sleep hours per day for caregiver (>8h)										
<6h	1.56 (1.09 to 2.03)	<0.001	-0.35 (-0.75 to 0.06)	0.09	0.88 (0.3 to 1.46)	0.003	1.27 (0.92 to 1.62)	<0.001	0.88 (0.46 to 1.29)	<0.001
6-8h	-0.1 (-0.49 to 0.29)	0.619	-0.49 (-0.85 to -0.12)	0.01	-0.87 (-1.37 to -0.37)	<0.001	1.18 (0.88 to 1.49)	<0.001	-0.07 (-0.44 to 0.29)	0.697
Co-caregivers (vs No)										
Yes	-0.87 (-1.11 to -0.64)	<0.001	-0.72 (-0.94 to -0.5)	<0.001	-3.13 (-3.41 to -2.86)	<0.001	0.31 (0.09 to 0.53)	0.006	-1.33 (-1.55 to -1.11)	<0.001
Duration of Current Care Episode (days) (vs >10)										
<3	-0.7 (-1.07 to -0.33)	<0.001	-0.37 (-0.71 to -0.04)	0.029	-1.98 (-2.42 to -1.54)	<0.001	-0.3 (-0.61 to 0.02)	0.063	-0.32 (-0.66 to 0.03)	0.07
3-5	-0.2 (-0.53 to 0.13)	0.239	-0.36 (-0.65 to -0.08)	0.013	-0.53 (-0.94 to -0.11)	0.013	-0.12 (-0.39 to 0.15)	0.381	0.06 (-0.24 to 0.36)	0.701
6-9	-0.29 (-0.62 to 0.04)	0.088	-0.49 (-0.78 to -0.2)	<0.001	-0.43 (-0.86 to -0.01)	0.043	-0.17 (-0.44 to 0.1)	0.212	-0.35 (-0.65 to -0.05)	0.022
Work status (vs Employed)										
Unemployed	-0.28 (-0.7 to 0.13)	0.178	0.16 (-0.18 to 0.51)	0.357	-1.35 (-1.89 to -0.82)	<0.001	0.14 (-0.19 to 0.47)	0.394	-0.83 (-1.22 to -0.44)	<0.001

© 2025 Ju W et al. JAMA Health Forum.

Nursing care skills (vs Extremely and Moderately skilled)										
Not skilled at all	0.48 (0.25 to 0.71)	<0.001	-0.52 (-0.73 to -0.32)	<0.001	1.06 (0.78 to 1.33)	<0.001	0.05 (-0.15 to 0.24)	0.637	-0.3 (-0.51 to -0.09)	0.004
Prevalence year (vs ≥5)										
<1	0.36 (0.03 to 0.68)	0.032	0.39 (0.1 to 0.69)	0.01	0.37 (-0.01 to 0.75)	0.054	0.37 (0.09 to 0.65)	0.01	0.12 (-0.19 to 0.43)	0.456
1-2.9	0.43 (0.09 to 0.77)	0.013	0.49 (0.18 to 0.8)	0.002	0.54 (0.15 to 0.94)	0.007	0.36 (0.07 to 0.65)	0.015	-0.13 (-0.45 to 0.19)	0.415
3-4.9	0.37 (-0.04 to 0.77)	0.078	0.45 (0.08 to 0.82)	0.018	0.26 (-0.22 to 0.74)	0.283	0.19 (-0.15 to 0.53)	0.278	-0.13 (-0.51 to 0.25)	0.49
Stage (vs IV)										
0	-0.42 (-1.43 to 0.6)	0.421	-0.19 (-1.12 to 0.74)	0.69	-0.21 (-1.57 to 1.15)	0.765	-0.11 (-0.99 to 0.76)	0.799	0.13 (-0.86 to 1.12)	0.794
I	-0.2 (-0.71 to 0.31)	0.435	0.28 (-0.2 to 0.76)	0.256	-0.27 (-0.89 to 0.35)	0.397	-0.18 (-0.62 to 0.26)	0.424	0.03 (-0.46 to 0.51)	0.91
II	-0.19 (-0.54 to 0.17)	0.306	-0.07 (-0.39 to 0.25)	0.683	0.2 (-0.24 to 0.63)	0.376	-0.45 (-0.74 to -0.16)	0.003	0.32 (-0.01 to 0.66)	0.06
III	-0.11 (-0.37 to 0.15)	0.403	0.09 (-0.14 to 0.32)	0.434	0.31 (-0.01 to 0.63)	0.062	-0.1 (-0.32 to 0.11)	0.353	-0.13 (-0.36 to 0.11)	0.297
Surgery (vs No)										
Yes	-1.45 (-1.93 to -0.96)	<0.001	-0.65 (-1.13 to -0.17)	0.008	-0.82 (-1.48 to -0.17)	0.014	-0.38 (-0.83 to 0.06)	0.091	-1.53 (-1.98 to -1.08)	<0.001
NRS2002 (vs <3)										
≥3	0.34 (0.1 to 0.57)	0.006	0.01 (-0.2 to 0.22)	0.909	1.12 (0.83 to 1.42)	<0.001	0.04 (-0.16 to 0.23)	0.71	0.1 (-0.11 to 0.32)	0.353
EQ-5D-5L value	-0.54 (-1.12 to 0.03)	0.063	0.49 (0.02 to 0.96)	0.043	1.26 (0.64 to 1.87)	<0.001	-0.63 (-1.13 to -0.13)	0.013	0.56 (0.07 to 1.04)	0.025

© 2025 Ju W et al. JAMA Health Forum.

eTable 4C The generalized linear model for factors associated with total scores of each subscale of CRA for adult-child caregivers.

	Heath		Self-esteem		Scheduling		Finance		Family support	
	Coefficient (95% CI)	p-value	Coefficient (95% CI)	p-value	Coefficient (95% CI)	p-value	Coefficient (95% CI)	p-value	Coefficient (95% CI)	p-value
Intercept	9.11 (8.28 to 9.93)	<0.001	19.68 (18.68 to 20.68)	<0.001	13.42 (12.23 to 14.61)	<0.001	11.1 (10.27 to 11.92)	<0.001	11.84 (10.98 to 12.71)	<0.001
SES Score	-0.07 (-0.11 to -0.03)	<0.001	-0.19 (-0.24 to -0.14)	<0.001	0.02 (-0.04 to 0.08)	0.608	-0.17 (-0.21 to -0.13)	<0.001	-0.12 (-0.17 to -0.08)	<0.001
Sex (vs Male)										
Female	-0.15 (-0.33 to 0.04)	0.115	-0.41 (-0.63 to -0.19)	<0.001	-0.74 (-1 to -0.47)	<0.001	-0.16 (-0.34 to 0.02)	0.084	-0.31 (-0.51 to -0.11)	0.002
Age group (vs <60)										
≥60	-0.17 (-0.72 to 0.37)	0.532	0.83 (0.15 to 1.52)	0.017	-1.2 (-1.91 to -0.48)	0.001	-0.23 (-0.79 to 0.33)	0.418	-0.14 (-0.71 to 0.42)	0.617
Self-reported health problem (vs No)										
Yes	0.19 (-0.19 to 0.57)	0.329	-0.53 (-0.97 to -0.1)	0.016	0.4 (-0.15 to 0.95)	0.151	-0.38 (-0.74 to -0.03)	0.034	-0.19 (-0.58 to 0.2)	0.334
Sleep hours per day for caregiver (>8h)										
<6h	1.44 (1.07 to 1.8)	<0.001	0.29 (-0.16 to 0.74)	0.206	1.18 (0.64 to 1.71)	<0.001	0.63 (0.28 to 0.97)	<0.001	1.13 (0.74 to 1.53)	<0.001
6-8h	0.2 (-0.1 to 0.51)	0.194	-0.36 (-0.76 to 0.04)	0.079	0.41 (-0.06 to 0.88)	0.086	0.82 (0.52 to 1.13)	<0.001	0.17 (-0.17 to 0.51)	0.325
Co-caregivers (vs No)										
Yes	0.09 (-0.1 to 0.27)	0.366	-0.51 (-0.73 to -0.28)	<0.001	-0.38 (-0.66 to -0.11)	0.006	-0.13 (-0.32 to 0.05)	0.164	-0.4 (-0.6 to -0.19)	<0.001
Duration of Current Care Episode (days) (vs >10)										
<3	-1 (-1.33 to -0.66)	<0.001	-0.78 (-1.2 to -0.37)	<0.001	-0.85 (-1.34 to -0.37)	0.001	-0.49 (-0.83 to -0.15)	0.005	-0.79 (-1.16 to -0.42)	<0.001
3-5	-0.62 (-0.91 to -0.33)	<0.001	-0.49 (-0.84 to -0.14)	0.006	-0.47 (-0.88 to -0.06)	0.026	-0.61 (-0.9 to -0.32)	<0.001	-0.54 (-0.86 to -0.23)	<0.001
6-9	-0.25 (-0.55 to 0.04)	0.089	-0.55 (-0.9 to -0.2)	0.002	0.33 (-0.09 to 0.76)	0.124	-0.41 (-0.71 to -0.12)	0.006	-0.63 (-0.94 to -0.32)	<0.001
Work status (vs Employed)										

© 2025 Ju W et al. JAMA Health Forum.

Unemployed	-0.11 (-0.37 to 0.15)	0.39	-0.53 (-0.84 to -0.21)	0.001	-0.35 (-0.72 to 0.02)	0.066	0.13 (-0.14 to 0.4)	0.342	-0.51 (-0.79 to -0.23)	<0.001
Nursing care skills (vs Extremely and Moderately skilled)										
Not skilled at all	-0.35 (-0.54 to -0.15)	<0.001	-1.06 (-1.29 to -0.83)	<0.001	0.17 (-0.11 to 0.45)	0.243	0.01 (-0.17 to 0.2)	0.878	-0.93 (-1.14 to -0.72)	<0.001
Prevalence year (vs ≥5)										
<1	0.04 (-0.33 to 0.4)	0.845	-0.16 (-0.61 to 0.29)	0.487	1.03 (0.52 to 1.53)	<0.001	0.31 (-0.05 to 0.67)	0.095	0.11 (-0.29 to 0.5)	0.6
1-2.9	-0.26 (-0.65 to 0.12)	0.18	-0.19 (-0.67 to 0.29)	0.441	0.2 (-0.33 to 0.74)	0.46	-0.03 (-0.42 to 0.35)	0.859	-0.13 (-0.55 to 0.29)	0.538
3-4.9	-0.15 (-0.63 to 0.33)	0.542	-0.67 (-1.26 to -0.08)	0.027	-0.04 (-0.71 to 0.63)	0.9	-0.24 (-0.71 to 0.24)	0.329	-0.33 (-0.85 to 0.19)	0.212
Stage (vs IV)										
0	-0.43 (-1.11 to 0.25)	0.212	0.56 (-0.36 to 1.49)	0.232	-0.49 (-1.53 to 0.55)	0.356	-0.23 (-0.98 to 0.52)	0.545	-0.06 (-0.86 to 0.74)	0.89
I	-0.25 (-0.64 to 0.14)	0.212	0.59 (0.07 to 1.11)	0.027	-0.31 (-0.9 to 0.27)	0.293	-0.25 (-0.66 to 0.15)	0.224	0.26 (-0.2 to 0.72)	0.273
II	0.08 (-0.25 to 0.41)	0.625	0.4 (-0.01 to 0.81)	0.055	-0.26 (-0.74 to 0.22)	0.282	-0.23 (-0.55 to 0.1)	0.176	0.23 (-0.13 to 0.6)	0.21
III	-0.04 (-0.25 to 0.17)	0.709	-0.14 (-0.4 to 0.11)	0.271	0.05 (-0.26 to 0.36)	0.741	-0.43 (-0.64 to -0.22)	<0.001	-0.09 (-0.32 to 0.14)	0.448
Surgery (vs No)										
Yes	-1.14 (-1.47 to -0.82)	<0.001	-0.31 (-0.75 to 0.12)	0.159	-1.05 (-1.56 to -0.55)	<0.001	-0.21 (-0.56 to 0.15)	0.257	-1.33 (-1.69 to -0.97)	<0.001
NRS2002 (vs <3)										
≥3	0.1 (-0.1 to 0.29)	0.32	-0.14 (-0.37 to 0.1)	0.246	0.75 (0.46 to 1.04)	<0.001	-0.09 (-0.28 to 0.1)	0.347	-0.11 (-0.33 to 0.1)	0.286
EQ-5D-5L value	-0.43 (-0.88 to 0.03)	0.068	0.44 (-0.07 to 0.94)	0.089	-1.36 (-2.02 to -0.7)	<0.001	-0.83 (-1.28 to -0.38)	<0.001	1.02 (0.58 to 1.46)	<0.001

© 2025 Ju W et al. JAMA Health Forum.

eTable 5. The sensitivity analysis further adjusting for the Social Deprivation Index (SDI) in the generalized linear models.

	Heath		Self-esteem		Scheduling		Finance		Family support	
	Coefficient (95% CI)	p-value	Coefficient (95% CI)	p-value	Coefficient (95% CI)	p-value	Coefficient (95% CI)	p-value	Coefficient (95% CI)	p-value
Intercept	5.75 (5.06 to 6.44)	<0.001	15.97 (15.21 to 16.73)	<0.001	12.22 (11.24 to 13.21)	<0.001	9.65 (8.99 to 10.31)	<0.001	8.7 (8.02 to 9.38)	<0.001
SES Score	-0.07 (-0.09 to -0.04)	<0.001	-0.14 (-0.17 to -0.11)	<0.001	-0.06 (-0.1 to -0.02)	0.003	-0.14 (-0.17 to -0.11)	<0.001	-0.13 (-0.15 to -0.1)	<0.001
Sex (vs Male)										
Female	0.18 (0.04 to 0.31)	0.011	-0.13 (-0.27 to 0.02)	0.09	-0.27 (-0.46 to -0.07)	0.007	0.02 (-0.11 to 0.14)	0.812	-0.08 (-0.22 to 0.05)	0.231
Age group (vs <60)										
≥60	0.72 (0.53 to 0.91)	<0.001	0.54 (0.34 to 0.74)	<0.001	-0.57 (-0.84 to -0.31)	<0.001	0.1 (-0.08 to 0.28)	0.266	-0.08 (-0.26 to 0.1)	0.379
Self-reported health problem (vs No)										
Yes	0.83 (0.61 to 1.04)	<0.001	-0.26 (-0.48 to -0.05)	0.016	0.92 (0.63 to 1.22)	<0.001	-0.1 (-0.28 to 0.09)	0.311	0.28 (0.08 to 0.48)	0.006
Sleep hours per day for caregiver (>8h)										
<6h	1.66 (1.38 to 1.93)	<0.001	0.2 (-0.1 to 0.5)	0.191	1.26 (0.87 to 1.66)	<0.001	0.85 (0.61 to 1.09)	<0.001	1.28 (1.01 to 1.55)	<0.001
6-8h	0.21 (-0.02 to 0.44)	0.073	-0.23 (-0.5 to 0.03)	0.087	-0.09 (-0.43 to 0.26)	0.62	0.94 (0.73 to 1.16)	<0.001	0.24 (0 to 0.48)	0.046
Co-caregivers (vs No)										
Yes	-0.42 (-0.56 to -0.28)	<0.001	-0.67 (-0.83 to -0.52)	<0.001	-1.76 (-1.96 to -1.55)	<0.001	-0.03 (-0.16 to 0.11)	0.705	-0.95 (-1.1 to -0.81)	<0.001
Duration of Current Care Episode (days) (vs ≥10)										
<3	-0.92 (-1.16 to -0.69)	<0.001	-0.64 (-0.9 to -0.38)	<0.001	-1.73 (-2.06 to -1.4)	<0.001	-0.4 (-0.63 to -0.18)	0.001	-0.66 (-0.9 to -0.42)	<0.001
3-5	-0.28 (-0.49 to -0.07)	0.009	-0.29 (-0.52 to -0.07)	0.01	-0.49 (-0.79 to -0.2)	0.001	-0.41 (-0.6 to -0.22)	<0.001	-0.05 (-0.26 to 0.16)	0.626
6-9	-0.22 (-0.43 to -0.01)	0.04	-0.5 (-0.72 to -0.27)	<0.001	-0.02 (-0.32 to 0.29)	0.905	-0.27 (-0.46 to -0.07)	0.007	-0.42 (-0.62 to -0.21)	<0.001
Work status (vs Employed)										
Unemployed	0 (-0.19 to 0.19)	0.993	-0.09 (-0.3 to 0.11)	0.37	-1.17 (-1.44 to -0.89)	<0.001	0.23 (0.05 to 0.41)	0.011	-0.68 (-0.86 to -0.49)	<0.001

© 2025 Ju W et al. JAMA Health Forum.

Nursing care skills (vs Extremely and Moderately skilled)										
Not skilled at all	0.16 (0.02 to 0.3)	0.026	-0.64 (-0.79 to -0.48)	<0.001	0.77 (0.57 to 0.97)	<0.001	0.06 (-0.07 to 0.19)	0.387	-0.44 (-0.58 to -0.3)	<0.001
Prevalence year (vs ≥5)										
<1	0.36 (0.13 to 0.59)	0.002	0.23 (-0.03 to 0.48)	0.078	1.1 (0.79 to 1.4)	<0.001	0.35 (0.13 to 0.57)	0.002	0.2 (-0.04 to 0.43)	0.098
1-2.9	0.23 (-0.01 to 0.47)	0.065	0.3 (0.04 to 0.57)	0.027	0.68 (0.36 to 1)	<0.001	0.16 (-0.07 to 0.39)	0.171	-0.05 (-0.29 to 0.2)	0.706
3-4.9	0.22 (-0.07 to 0.52)	0.14	0.04 (-0.29 to 0.36)	0.82	0.45 (0.06 to 0.85)	0.025	-0.01 (-0.29 to 0.27)	0.951	-0.16 (-0.46 to 0.14)	0.292
Stage (vs IV)										
0	-0.35 (-0.9 to 0.2)	0.211	0.38 (-0.28 to 1.04)	0.257	-0.42 (-1.26 to 0.43)	0.335	-0.22 (-0.78 to 0.34)	0.44	0.03 (-0.58 to 0.63)	0.933
I	-0.26 (-0.56 to 0.03)	0.081	0.39 (0.04 to 0.75)	0.028	-0.1 (-0.53 to 0.34)	0.653	-0.35 (-0.64 to -0.06)	0.018	0.18 (-0.14 to 0.5)	0.269
II	-0.13 (-0.36 to 0.1)	0.268	0.09 (-0.16 to 0.35)	0.48	-0.02 (-0.35 to 0.31)	0.913	-0.39 (-0.6 to -0.18)	0	0.21 (-0.02 to 0.45)	0.074
III	-0.12 (-0.28 to 0.04)	0.133	-0.11 (-0.28 to 0.06)	0.219	0.25 (0.02 to 0.47)	0.035	-0.34 (-0.48 to -0.19)	<0.001	-0.11 (-0.27 to 0.05)	0.168
Surgery (vs No)										
Yes	-0.9 (-1.16 to -0.64)	<0.001	-0.05 (-0.37 to 0.27)	0.753	-1.02 (-1.42 to -0.61)	<0.001	-0.1 (-0.37 to 0.17)	0.474	-1.01 (-1.28 to -0.74)	<0.001
NRS2002 (vs <3)										
≥3	0.29 (0.14 to 0.43)	<0.001	0.02 (-0.14 to 0.17)	0.818	0.98 (0.77 to 1.2)	<0.001	-0.07 (-0.2 to 0.07)	0.319	0.09 (-0.06 to 0.23)	0.229
EQ-5D-5L value	-0.32 (-0.65 to 0.01)	0.058	0.51 (0.17 to 0.85)	0.003	0.09 (-0.36 to 0.54)	0.696	-0.72 (-1.04 to -0.39)	<0.001	0.87 (0.57 to 1.17)	<0.001
SDI	7.08 (6.09 to 8.06)	<0.001	6.95 (5.85 to 8.05)	<0.001	4.71 (3.26 to 6.16)	<0.001	0.76 (-0.21 to 1.74)	0.124	8.54 (7.54 to 9.54)	<0.001

© 2025 Ju W et al. JAMA Health Forum.

eTable 6A. Associations of educational level with the score of CRA-subscale stratified by household income level

CRA-subscale	Income level	Educational level	β (95% CI)	p-value	P for interaction
Heath	Overall	Tertiary	Reference	-	0.269
		Primary	0 (-0.24 to 0.23)	0.97	
		Secondary	0.01 (-0.22 to 0.24)	0.942	
	Quartile 1 (<20000 CNY)	Tertiary	Reference	-	
		Primary	1.25 (-0.4 to 2.89)	0.138	
		Secondary	1.38 (-0.3 to 3.06)	0.108	
	Quartile 2 (20000-59999 CNY)	Tertiary	Reference	-	
		Primary	0.37 (-0.41 to 1.16)	0.35	
		Secondary	0.44 (-0.36 to 1.24)	0.284	
	Quartile 3 (60000-99999 CNY)	Tertiary	Reference	-	
		Primary	0.11 (-0.39 to 0.62)	0.658	
		Secondary	0.16 (-0.35 to 0.68)	0.529	
	Quartile 4 (≥100000 CNY)	Tertiary	Reference	-	
		Primary	-0.41 (-0.76 to -0.05)	0.024	
		Secondary	-0.18 (-0.49 to 0.12)	0.24	
Self-esteem	Overall	Tertiary	Reference	-	0.252
		Primary	0.89 (0.63 to 1.14)	<0.001	
		Secondary	0.6 (0.35 to 0.86)	<0.001	
	Quartile 1 (<20000 CNY)	Tertiary	Reference	-	
		Primary	0.2 (-1.53 to 1.93)	0.822	
		Secondary	-0.2 (-1.97 to 1.58)	0.829	
	Quartile 2 (20000-59999 CNY)	Tertiary	Reference	-	
		Primary	-0.12 (-1.04 to 0.8)	0.803	
		Secondary	-0.33 (-1.27 to 0.61)	0.493	
	Quartile 3 (60000-99999 CNY)	Tertiary	Reference	-	
		Primary	1.24 (0.7 to 1.77)	<0.001	

© 2025 Ju W et al. JAMA Health Forum.

		Secondary	0.94 (0.4 to 1.48)	0.001	
	Quartile 4 (≥100000 CNY)	Tertiary	Reference	-	
		Primary	0.21 (-0.17 to 0.59)	0.272	
		Secondary	0.25 (-0.07 to 0.58)	0.125	
Scheduling	Overall	Tertiary	Reference	-	0.087
		Primary	0.16 (-0.14 to 0.47)	0.298	
		Secondary	-0.1 (-0.4 to 0.21)	0.537	
	Quartile 1 (<20000 CNY)	Tertiary	Reference	-	
		Primary	2.09 (0.29 to 3.89)	0.023	
		Secondary	2.13 (0.29 to 3.97)	0.023	
	Quartile 2 (20000-59999 CNY)	Tertiary	Reference	-	
		Primary	1.17 (0.25 to 2.09)	0.013	
		Secondary	1.21 (0.27 to 2.15)	0.012	
	Quartile 3 (60000-99999 CNY)	Tertiary	Reference	-	
		Primary	0.3 (-0.37 to 0.96)	0.381	
		Secondary	-0.14 (-0.81 to 0.53)	0.679	
	Quartile 4 (≥100000 CNY)	Tertiary	Reference	-	
		Primary	0.77 (0.26 to 1.27)	0.003	
		Secondary	0.32 (-0.11 to 0.75)	0.143	
Finance	Overall	Tertiary	Reference	-	0.017
		Primary	1.09 (0.89 to 1.28)	<0.001	
		Secondary	0.76 (0.57 to 0.95)	<0.001	
	Quartile 1 (<20000 CNY)	Tertiary	Reference	-	
		Primary	1.03 (-0.21 to 2.27)	0.103	
		Secondary	0.85 (-0.42 to 2.12)	0.188	
	Quartile 2 (20000-59999 CNY)	Tertiary	Reference	-	
		Primary	1.3 (0.64 to 1.96)	<0.001	
		Secondary	1.24 (0.56 to 1.91)	<0.001	

© 2025 Ju W et al. JAMA Health Forum.

	Quartile 3 (60000-99999 CNY)	Tertiary	Reference	-	
		Primary	1.16 (0.78 to 1.55)	<0.001	
		Secondary	0.73 (0.34 to 1.11)	<0.001	
	Quartile 4 (≥100000 CNY)	Tertiary	Reference	-	
		Primary	0.76 (0.45 to 1.08)	<0.001	
		Secondary	0.42 (0.14 to 0.69)	0.003	
Family support	Overall	Tertiary	Reference	-	<0.001
		Primary	0.09 (-0.16 to 0.33)	0.489	
		Secondary	0.15 (-0.09 to 0.39)	0.216	
	Quartile 1 (<20000 CNY)	Tertiary	Reference	-	
		Primary	2.04 (0.38 to 3.7)	0.016	
		Secondary	1.85 (0.15 to 3.54)	0.033	
	Quartile 2 (20000-59999 CNY)	Tertiary	Reference	-	
		Primary	0.65 (-0.13 to 1.42)	0.103	
		Secondary	0.59 (-0.21 to 1.39)	0.147	
	Quartile 3 (60000-99999 CNY)	Tertiary	Reference	-	
		Primary	0.12 (-0.38 to 0.61)	0.648	
		Secondary	0.31 (-0.19 to 0.81)	0.227	
	Quartile 4 (≥100000 CNY)	Tertiary	Reference	-	
		Primary	-0.88 (-1.26 to -0.49)	<0.001	
		Secondary	-0.12 (-0.45 to 0.21)	0.476	

© 2025 Ju W et al. JAMA Health Forum.

eTable 6B. Associations of household income level with the score of CRA-subscale stratified by educational level

CRA-subscale	Educational level	Income level	β (95% CI)	p-value	P for interaction
Heath	Overall	Quartile 4 (≥100000 CNY)	Reference	-	0.286
		Quartile 1 (<20000 CNY)	0.61 (0.37 to 0.86)	<0.001	
		Quartile 2 (20000-59999 CNY)	0.22 (0.03 to 0.4)	0.02	
		Quartile 3 (60000-99999 CNY)	0.11 (-0.06 to 0.27)	0.208	
	Primary	Quartile 4 (≥100000 CNY)	Reference	-	
		Quartile 1 (<20000 CNY)	0.62 (0.31 to 0.94)	<0.001	
		Quartile 2 (20000-59999 CNY)	0.23 (-0.04 to 0.5)	0.089	
		Quartile 3 (60000-99999 CNY)	0.13 (-0.13 to 0.38)	0.341	
	Secondary	Quartile 4 (≥100000 CNY)	Reference	-	
		Quartile 1 (<20000 CNY)	0.67 (0.15 to 1.19)	0.012	
		Quartile 2 (20000-59999 CNY)	0.34 (0.02 to 0.66)	0.035	
		Quartile 3 (60000-99999 CNY)	0.19 (-0.07 to 0.45)	0.148	
	Tertiary	Quartile 4 (≥100000 CNY)	Reference	-	
		Quartile 1 (<20000 CNY)	-1.26 (-2.94 to 0.42)	0.143	
		Quartile 2 (20000-59999 CNY)	-0.31 (-1.13 to 0.5)	0.447	
		Quartile 3 (60000-99999 CNY)	0.02 (-0.52 to 0.55)	0.952	
Self-esteem	Overall	Quartile 4 (≥100000 CNY)	Reference	-	0.252
		Quartile 1 (<20000 CNY)	0.67 (0.4 to 0.94)	<0.001	
		Quartile 2 (20000-59999 CNY)	0.71 (0.51 to 0.91)	<0.001	
		Quartile 3 (60000-99999 CNY)	0.66 (0.48 to 0.84)	<0.001	
	Primary	Quartile 4 (⩾100000 CNY)	Reference	-	
		Quartile 1 (<20000 CNY)	0.62 (0.29 to 0.95)	<0.001	

© 2025 Ju W et al. JAMA Health Forum.

		Quartile 2 (20000-59999 CNY)	0.54 (0.26 to 0.82)	<0.001	
		Quartile 3 (60000-99999 CNY)	0.52 (0.25 to 0.79)	<0.001	
	Secondary	Quartile 4 (≥100000 CNY)	Reference	-	
		Quartile 1 (<20000 CNY)	0.07 (-0.53 to 0.66)	0.828	
		Quartile 2 (20000-59999 CNY)	0.31 (-0.05 to 0.68)	0.096	
		Quartile 3 (60000-99999 CNY)	0.51 (0.21 to 0.81)	0.001	
	Tertiary	Quartile 4 (≥100000 CNY)	Reference	-	
		Quartile 1 (<20000 CNY)	0.25 (-1.74 to 2.23)	0.808	
		Quartile 2 (20000-59999 CNY)	1.19 (0.23 to 2.15)	0.015	
		Quartile 3 (60000-99999 CNY)	0.36 (-0.27 to 1)	0.257	
Scheduling	Overall	Quartile 4 (≥100000 CNY)	Reference	-	0.087
		Quartile 1 (<20000 CNY)	0.12 (-0.2 to 0.44)	0.469	
		Quartile 2 (20000-59999 CNY)	-0.33 (-0.57 to -0.09)	0.008	
		Quartile 3 (60000-99999 CNY)	-0.54 (-0.76 to -0.33)	<0.001	
	Primary	Quartile 4 (≥100000 CNY)	Reference	-	
		Quartile 1 (<20000 CNY)	-0.09 (-0.46 to 0.28)	0.621	
		Quartile 2 (20000-59999 CNY)	-0.39 (-0.7 to -0.07)	0.016	
		Quartile 3 (60000-99999 CNY)	-0.53 (-0.84 to -0.22)	0.001	
	Secondary	Quartile 4 (≥100000 CNY)	Reference	-	
		Quartile 1 (<20000 CNY)	0.37 (-0.37 to 1.12)	0.328	
		Quartile 2 (20000-59999 CNY)	-0.31 (-0.76 to 0.15)	0.19	
		Quartile 3 (60000-99999 CNY)	-0.66 (-1.04 to -0.29)	<0.001	
	Tertiary	Quartile 4 (≥100000 CNY)	Reference	-	
		Quartile 1 (<20000 CNY)	-1.5 (-4.17 to 1.17)	0.272	
		Quartile 2 (20000-59999 CNY)	-1.33 (-2.62 to -0.04)	0.044	
		Quartile 3 (60000-99999 CNY)	-0.13 (-0.98 to 0.72)	0.761	

© 2025 Ju W et al. JAMA Health Forum.

Finance	Overall	Quartile 4 (≥100000 CNY)	Reference	-	0.017
		Quartile 1 (<20000 CNY)	0.68 (0.48 to 0.89)	<0.001	
		Quartile 2 (20000-59999 CNY)	0.41 (0.25 to 0.56)	<0.001	
		Quartile 3 (60000-99999 CNY)	0.62 (0.48 to 0.76)	<0.001	
	Primary	Quartile 4 (≥100000 CNY)	Reference	-	
		Quartile 1 (<20000 CNY)	0.42 (0.17 to 0.67)	0.001	
		Quartile 2 (20000-59999 CNY)	0.05 (-0.16 to 0.25)	0.674	
		Quartile 3 (60000-99999 CNY)	0.51 (0.31 to 0.71)	<0.001	
	Secondary	Quartile 4 (≥100000 CNY)	Reference	-	
		Quartile 1 (<20000 CNY)	0.8 (0.35 to 1.25)	0.001	
		Quartile 2 (20000-59999 CNY)	0.65 (0.37 to 0.92)	<0.001	
		Quartile 3 (60000-99999 CNY)	0.49 (0.26 to 0.71)	<0.001	
	Tertiary	Quartile 4 (≥100000 CNY)	Reference	-	
		Quartile 1 (<20000 CNY)	-0.13 (-1.71 to 1.45)	0.87	
		Quartile 2 (20000-59999 CNY)	-0.33 (-1.09 to 0.43)	0.393	
		Quartile 3 (60000-99999 CNY)	0.28 (-0.22 to 0.78)	0.279	
Family support	Overall	Quartile 4 (≥100000 CNY)	Reference	-	<0.001
		Quartile 1 (<20000 CNY)	1.3 (1.05 to 1.56)	<0.001	
		Quartile 2 (20000-59999 CNY)	0.96 (0.77 to 1.15)	<0.001	
		Quartile 3 (60000-99999 CNY)	0.43 (0.26 to 0.6)	<0.001	
	Primary	Quartile 4 (≥100000 CNY)	Reference	-	
		Quartile 1 (<20000 CNY)	1.69 (1.39 to 1.99)	<0.001	
		Quartile 2 (20000-59999 CNY)	1.37 (1.12 to 1.63)	<0.001	
		Quartile 3 (60000-99999 CNY)	0.73 (0.48 to 0.98)	<0.001	
	Secondary	Quartile 4 (≥100000 CNY)	Reference	-	
		Quartile 1 (<20000 CNY)	0.87 (0.31 to 1.44)	0.003	

© 2025 Ju W et al. JAMA Health Forum.

	Quartile 2 (20000-59999 CNY)	0.56 (0.21 to 0.9)	0.002
	Quartile 3 (60000-99999 CNY)	0.33 (0.05 to 0.61)	0.022
	Quartile 4 (≥100000 CNY)	Reference	-
Tertiary	Quartile 1 (<20000 CNY)	-1.02 (-3.01 to 0.97)	0.317
	Quartile 2 (20000-59999 CNY)	0.24 (-0.72 to 1.2)	0.624
	Quartile 3 (60000-99999 CNY)	0.36 (-0.28 to 0.99)	0.267

© 2025 Ju W et al. JAMA Health Forum.

eTable 7 The proportions of caregivers with the anxiety and depression symptom by SES quartiles

	Overall	SES quartiles				P-value
		Lowest	Lower-middle	Upper-middle	Highest	
**All caregivers**						
**GAD-7**						
≥5	2473 (37.2%)	813 (43.4%)	611 (35.1%)	430 (32.6%)	619 (36.2%)	
<5	4156 (62.6%)	1056 (56.4%)	1124 (64.6%)	888 (67.3%)	1088 (63.6%)	<0.001
missing	14 (0.2%)	3 (0.2%)	4 (0.3%)	2 (0.1%)	5 (0.2%)	
**PHQ-9**						
≥5	1814 (26.7%)	648 (34.6%)	403 (23.2%)	283 (21.4%)	443 (25.9%)	
<5	4919 (72.9%)	1220 (65.2%)	1330 (76.5%)	1033 (78.3%)	1263 (73.8%)	<0.001
missing	20 (0.4%)	4 (0.2%)	6 (0.3%)	4 (0.3%)	6 (0.3%)	
**Spouse caregivers**						
**GAD-7**						
≥5	976 (34.7%)	489 (43.5%)	251 (31.7%)	138 (27.9%)	95 (24%)	
<5	1832 (65.1%)	636 (56.5%)	540 (68.2%)	355 (71.9%)	299 (75.5%)	<0.001
missing	4 (0.1%)	0	1 (0.1%)	1 (0.2%)	2 (0.5%)	
**PHQ-9**						
≥5	687 (24.4%)	389 (34.6%)	155 (19.6%)	90 (18.2%)	51 (12.9%)	
<5	2120 (75.4%)	736 (65.4%)	636 (80.3%)	402 (81.4%)	343 (86.6%)	<0.001
missing	5 (0.2%)	0	1 (0.1%)	2 (0.4%)	2 (0.5%)	
**Adult-child caregivers**						
**GAD-7**						
≥5	1393 (40.9%)	288 (44.7%)	339 (40.6%)	266 (36.8%)	496 (41.6%)	
<5	2002 (58.8%)	355 (55.0%)	493 (59.0%)	455 (63.0%)	694 (58.2%)	0.309
missing	9 (0.3%)	2 (0.3%)	3 (0.4%)	1 (0.1%)	3 (0.3%)	
**PHQ-9**						
≥5	1018 (29.9%)	229 (35.5%)	232 (27.8%)	175 (24.2%)	380 (31.9%)	
<5	2372 (69.7%)	413 (64.0%)	598 (71.6%)	545 (75.5%)	809 (67.8%)	0.44
missing	14 (0.4%)	3 (0.5%)	5 (0.6%)	2 (0.3%)	4 (0.3%)	

GAD-7= Generalized Anxiety Disorder scale 7; PHQ-9= Patient Health Questionnaire 9

The p-value was estimated by Cochrane-Armitage Test

© 2025 Ju W et al. JAMA Health Forum.

eTable 8. The associations between SES status and depression anxiety and symptom in the sensitivity analysis further adjusting for the SDI

	Psychological risk					
	**All caregivers**	**p-value**	**Spouse caregivers**	**p-value**	**Adult-child caregiver**	**p-value**
**PHQ-9⩾5**						
SES group						
Lowest	1.52 (1.26 to 1.83)	<0.001	2.42 (1.68 to 3.47)	<0.001	1.12 (0.86 to 1.47)	0.402
Lower-middle	0.86 (0.72 to 1.04)	0.11	1.23 (0.84 to 1.8)	0.289	0.83 (0.65 to 1.06)	0.136
Upper-middle	0.75 (0.62 to 0.92)	0.004	1.13 (0.75 to 1.71)	0.56	0.7 (0.54 to 0.89)	0.005
Highest	Reference	-	Reference	-	Reference	-
**GAD-7≥5**						
SES group						
Lowest	1.24 (1.04 to 1.47)	0.017	1.48 (1.1 to 1.99)	0.01	1.06 (0.82 to 1.37)	0.651
Lower-middle	0.93 (0.79 to 1.09)	0.37	1.1 (0.81 to 1.51)	0.534	1 (0.8 to 1.25)	0.986
Upper-middle	0.82 (0.69 to 0.98)	0.026	1 (0.71 to 1.4)	0.993	0.84 (0.66 to 1.05)	0.125
Highest	Reference	-	Reference	-	Reference	-

© 2025 Ju W et al. JAMA Health Forum.

eTable 9A. Association of household income level with the risk of depression and anxiety symptom stratified by educational level

Educational level	Income level	Depression symptom (PHQ-9≥5)			Anxiety symptom (GAD-7≥5)		
		OR (95% CI)	p-value	P for interaction	OR (95% CI)	p-value	P for interaction
Primary	Quartile 4 (≥100000 CNY)	Reference	-	0.007	Reference	-	<0.001
	Quartile 1 (<20000 CNY)	2.04 (0.37 to 11.13)	0.409		0.55 (0.11 to 2.76)	0.467	
	Quartile 2 (20000-59999 CNY)	0.79 (0.36 to 1.73)	0.56		0.61 (0.27 to 1.38)	0.236	
	Quartile 3 (60000-99999 CNY)	0.99 (0.6 to 1.65)	0.984		0.8 (0.47 to 1.35)	0.4	
Secondary	Quartile 4 (≥100000 CNY)	Reference	-		Reference	-	
	Quartile 1 (<20000 CNY)	2.96 (2.25 to 3.91)	<0.001		5.76 (4.13 to 8.03)	<0.001	
	Quartile 2 (20000-59999 CNY)	1.67 (1.31 to 2.13)	<0.001		2.83 (2.09 to 3.82)	<0.001	
	Quartile 3 (60000-99999 CNY)	1.46 (1.15 to 1.85)	0.002		1.69 (1.25 to 2.29)	0.001	
Tertiary	Quartile 4 (≥100000 CNY)	Reference	-		Reference	-	
	Quartile 1 (<20000 CNY)	1.82 (1.14 to 2.91)	0.013		2.76 (1.69 to 4.52)	<0.001	
	Quartile 2 (20000-59999 CNY)	1.23 (0.92 to 1.65)	0.159		1.73 (1.27 to 2.37)	0.001	
	Quartile 3 (60000-99999 CNY)	0.84 (0.66 to 1.07)	0.166		0.93 (0.71 to 1.21)	0.579	

© 2025 Ju W et al. JAMA Health Forum.

eTable 9B. Association of educational level with the risk of depression and anxiety symptom stratified by household income level

Income level	Educational level	Depression symptom (PHQ-9≥5)			Anxiety symptom (GAD-7≥5)		
		OR (95% CI)	p-value	P for interaction	OR (95% CI)	p-value	P for interaction
Quartile 1 (<20000 CNY)	Tertiary	Reference	-	0.007	Reference	-	<0.001
	Primary	0.86 (0.2 to 3.83)	0.848		2.16 (0.46 to 10.1)	0.327	
	Secondary	0.77 (0.17 to 3.52)	0.737		2.33 (0.48 to 11.25)	0.291	
Quartile 2 (20000-59999 CNY)	Tertiary	Reference	-		Reference	-	
	Primary	0.96 (0.48 to 1.91)	0.901		1.07 (0.52 to 2.22)	0.848	
	Secondary	1.05 (0.52 to 2.13)	0.884		1.39 (0.66 to 2.93)	0.381	
Quartile 3 (60000-99999CNY)	Tertiary	Reference	-		Reference	-	
	Primary	0.74 (0.47 to 1.16)	0.195		0.77 (0.48 to 1.25)	0.292	
	Secondary	0.65 (0.41 to 1.02)	0.059		0.87 (0.54 to 1.41)	0.572	
Quartile 4 (≥100000 CNY)	Tertiary	Reference	-		Reference	-	
	Primary	0.47 (0.34 to 0.64)	<0.001		0.27 (0.19 to 0.4)	<0.001	
	Secondary	0.74 (0.57 to 0.96)	0.024		0.63 (0.48 to 0.84)	0.001	

© 2025 Ju W et al. JAMA Health Forum.

eTable 10 The direct caregiving-related expense for cancer patients in the past year.

	Total expense		Medical expense			Expense for traffic			Expense for meals and accommodation			Expense for nutrition			Expense for nursing staff		
	Mean	Median	Mean	Median	Percent[a] (%)	Mean	Median	Percent[a] (%)	Mean	Median	Percent[a] (%)	Mean	Median	Percent[a] (%)	Mean	Median	Percent[a] (%)
**Overall**	100694	88080	85310	70898	84.7	3758	2400	3.7	8393	6000	8.3	3060	0	3	173	0	0
**Age group**																	
60-69	105339	93000	89858	80000	85.3	3944	2400	3.7	8354	6000	7.9	3010	0	2.9	173	0	0
70-79	95916	83400	80542	70000	84	3617	2400	3.8	8412	6000	8.8	3141	0	3.3	203	0	0
⩾80	85534	73900	70954	60000	83	2868	1200	3.4	8633	6000	10.1	3063	0	3.6	15	0	0
**Cancer Site**																	
lung	102619	91960	86450	80000	84.2	4115	2400	4	8687	6000	8.5	3244	0	3.2	123	0	0
stomach	105032	92552	89718	80000	85.4	3654	2400	3.5	7882	6000	7.5	3404	0	3.2	374	0	0
esophagus	101115	84880	84634	68281	83.7	3704	2400	3.7	9706	7200	9.6	2859	0	2.8	213	0	0
colorectum	97136	83740	82378	70000	84.8	3362	2400	3.5	7531	6000	7.8	3760	0	3.9	104	0	0
liver	95812	85000	79603	70000	83.1	3905	2400	4.1	9081	6000	9.5	2839	0	3	384	0	0
breast	103503	89600	91208	80000	88.1	3357	2400	3.2	6422	4800	6.2	2502	0	2.4	14	0	0
others	98342	86760	83302	70000	84.7	3637	2400	3.7	8686	6000	8.8	2504	0	2.5	212	0	0
**Stage**																	
0 or I	87253	74280	72753	60000	83.4	3967	2400	4.5	8196	6000	9.4	2297	0	2.6	39	0	0
II	103638	88540	88914	76500	85.8	3508	2400	3.4	8102	6000	7.8	2915	0	2.8	200	0	0
III	102574	90800	86649	80000	84.5	3655	2400	3.6	8787	6000	8.6	3213	0	3.1	269	0	0
IV	99491	87200	84314	70000	84.7	3764	2400	3.8	8152	6000	8.2	3121	0	3.1	141	0	0
**HDI of hospital's location [b]**																	
Low	92567	76000	76817	60000	83	3937	1440	4.3	9182	6660	9.9	2455	0	2.7	176	0	0
Middle	103255	93200	88811	80000	86	3531	2400	3.4	8416	6000	8.2	2263	0	2.2	235	0	0
High	104712	90800	88225	80000	84.3	3935	2400	3.8	7579	4800	7.2	4899	0	4.7	75	0	0
**First-year diagnosis**																	
Yes	106851	92000	90090	80000	84.3	4331	2400	4.1	9103	6000	8.5	3138	0	2.9	189	0	0
No	92941	83420	79291	70000	85.3	3036	1800	3.3	7498	6000	8.1	2962	0	3.2	155	0	0

[a] the proportion of each item out of total expense

[b] HDI=High Development Index. The HDI was proposed by United Nations Development Program and is a composite measure of socioeconomic development including life expectancy, education, and gross income per capita index.

The low HDI regions (HDI thresholds:⩽0.708; ⩽25th percentile of HDI) include Guizhou, Qinghai Xizang, Yunnan, Anhui, Gansu, Guangxi and Sichuan

The middle HDI regions (HDI thresholds:(0.708-0.754]; >25th to 75th percentile of HDI) include Hainan, Hebei, Henan, Heilongjiang, Jiangxi, Ningxia, Shanxi, Xinjiang, Fujian, Hubei, Hunan, Jilin, Inner-Mongolia, Shandong, Shannxi, Chongqing

The high HDI regions (HDI thresholds:>0.754; >75th percentile of HDI) include Guangdong, Jiangsu, Liaoning, Zhejiang, Beijing, Shanghai, Tianjin

© 2025 Ju W et al. JAMA Health Forum.

eTable 11 The indirect economic burden for employed caregivers by SES quartiles

	Overall (n=3219)	SES quartiles				P-value
		Lowest (n=566)	Lower-middle (n=747)	Upper-middle (n=705)	Highest (n=1192)	
**Loss of work days per month**						
0	881 (27.4%)	206 (36.4%)	215 (28.8%)	176 (25.0%)	283 (23.7%)	
<3	553 (17.2%)	81 (14.3%)	112 (15.0%)	144 (20.4%)	212 (17.8%)	
3-5	939 (29.2%)	163 (28.8%)	219 (29.3%)	210 (29.8%)	344 (28.9%)	<0.001
6-9	574 (17.8%)	72 (12.7%)	145 (19.4%)	120 (17.0%)	237 (19.9%)	
⩾10	271 (8.4%)	44 (7.8%)	56 (7.5%)	55 (7.8%)	116 (9.7%)	
**Loss of income per month**						
0	1195 (37.1%)	234 (41.3%)	265 (35.5%)	247 (35.0%)	447 (37.5%)	
<500	486 (15.1%)	92 (16.3%)	147 (19.7%)	132 (18.7%)	113 (9.5%)	
500-999	489 (15.2%)	95 (16.8%)	130 (17.4%)	102 (14.5%)	159 (13.3%)	<0.001
1000-1499	485 (15.1%)	78 (13.8%)	120 (16.1%)	112 (15.9%)	175 (14.7%)	
⩾1500	563 (17.5%)	67 (11.8%)	85 (11.4%)	112 (15.9%)	298 (25.0%)	
**Proportion of loss of work days on total work days per month**						
0	791 (25.4%)	169 (32.4%)	193 (26.7%)	162 (23.5%)	266 (22.7%)	
<10%	447 (14.4%)	54 (10.3%)	82 (11.3%)	112 (16.3%)	195 (16.7%)	
10%-19.9%	745 (23.9%)	126 (24.1%)	197 (27.2%)	189 (27.4%)	231 (19.7%)	0.002
20%-29.9%	596 (19.1%)	80 (15.3%)	138 (19.1%)	126 (18.3%)	252 (21.5%)	
⩾30%	535 (17.2%)	93 (17.8%)	114 (15.8%)	100 (14.5%)	227 (19.4%)	
**Proportion of loss of income on total income per month**						
0	1195 (37.1%)	234 (41.3%)	265 (35.5%)	247 (35.0%)	447 (37.5%)	
<10%	618 (19.2%)	60 (10.6%)	136 (18.2%)	167 (23.7%)	251 (21.1%)	
10%-19.9%	613 (19.1%)	52 (9.2%)	138 (18.5%)	125 (17.7%)	297 (24.9%)	<0.001
20%-29.9%	390 (12.1%)	85 (15.0%)	109 (14.6%)	81 (11.5%)	115 (9.7%)	
⩾30%	402 (12.5%)	135 (23.9%)	99 (13.25%)	85 (12.1%)	82 (6.9%)	

© 2025 Ju W et al. JAMA Health Forum.
