# Supplementary material for: Socioeconomic Disparities in Caregiver Burden Among Families of Older Patients With Cancer
Source: JAMA Health Forum. 2025 Dec 26;6(12):e255614. doi: 10.1001/jamahealthforum.2025.5614 (PMC12743282; doi:10.1001/jamahealthforum.2025.5614)
Supplement: Supplement 2. — Data Sharing Statement [file jamahealthforum-e255614-s002.pdf]

Data Sharing Statement

Ju. Socioeconomic Disparities in Caregiver Burden Among Families of Older Patients With Cancer. *JAMA Health Forum*. Published December 26, 2025.
doi:10.1001/jamahealthforum.2025.5614

Data
Data available: No
Additional Information: Due to restrictions based on Chinese regulations and the current ethical approval for this study, the individual-level data used to generate the results in this article are not publicly available. Deidentified data that underlie the aggregate results reported in the article (eg, Tables) may be made available to qualified researchers on reasonable request to the corresponding author, subject to the execution of appropriate data use agreements.
